# Autism Spectrum Disorder, Attention‐Deficit/Hyperactivity Disorder, Tic Disorder, and Obsessive‐Compulsive Disorder in Individuals With Eating Disorders and Their Siblings: A Nationwide Finnish Cohort Study

**DOI:** 10.1002/eat.70171

**Published:** 2026-07-09

**Authors:** Emma Saure, Pyry N. Sipilä, Susanna Hinkka‐Yli‐Salomäki, Andre Sourander, Elina Santti, Anu Raevuori

**Affiliations:** ^1^ Clinicum, Department of Psychiatry University of Helsinki Helsinki Finland; ^2^ Department of Public Health University of Helsinki Helsinki Finland; ^3^ Research Centre for Child Psychiatry, INVEST Flagship University of Turku Turku Finland; ^4^ Department of Child Psychiatry Turku University Hospital Turku Finland; ^5^ Department of Psychiatry, Division of Adolescent Psychiatry Helsinki University Hospital and University of Helsinki Helsinki Finland

**Keywords:** attention deficit/hyperactivity disorder, autism Spectrum disorder, feeding and eating disorders, obsessive‐compulsive disorder, tic disorders

## Abstract

**Objective:**

Neurodevelopmental conditions frequently co‐occur with eating disorders (EDs). This study aimed to investigate the occurrence of attention‐deficit/hyperactivity disorder (ADHD), autism spectrum disorder (ASD), tic disorder (TD), and obsessive‐compulsive disorder (OCD) in individuals with EDs and their full and half siblings.

**Method:**

Study population comprised all individuals born in Finland between 1991 and 2001 with a recorded diagnosis of the following ICD‐10 EDs (*N* = 4240): anorexia nervosa (AN; F50.0/F50.1), bulimia nervosa (BN; F50.2/F50.3), and other/unspecified EDs (F50.8/F50.9). Population controls without ED (*N* = 16,494) and full and half siblings of participants with ED and controls (*N* = 36,301) were included. Conditional logistic regression was applied in analysis.

**Results:**

Individuals with AN had increased occurrence of ASD, ADHD, and OCD compared to population controls (Odds ratios [ORs]: 6.62 [95% CI 4.21–10.39]; 3.30 [95% CI 2.33–4.69]; 10.67 [95% CI 7.07–16.10], respectively). Individuals with other/unspecified EDs had increased occurrence of ASD, ADHD, TD, and OCD (ORs: 9.06 [95% CI 5.5–14.77]; 2.78 [95% CI 1.92–4.01]; 4.68 [95% CI 1.94–11.32]; 7.64 [95% CI 4.92–11.87], respectively). The occurrence for ASD was particularly pronounced in males with other/unspecified EDs (OR 32.40 [95% CI 9.78–107.39]). Compared to full siblings of population controls, full siblings of individuals with AN had two‐fold odds for ASD and three‐fold odds for OCD; full siblings of individuals with other/unspecified EDs had 2–3‐fold odds for ASD, ADHD, TD, and OCD.

**Discussion:**

Individuals with EDs have a high occurrence of neurodevelopmental conditions, also extending to their full siblings. These findings provide detailed characterization of comorbidity and the familial aggregation of these conditions.

## Summary

- Individuals with eating disorders are more likely than the general population to have autism spectrum disorder, attention‐deficit/hyperactivity disorder (ADHD), tic disorder, or obsessive‐compulsive disorder.
- Full siblings of individuals with anorexia nervosa or other/unspecified eating disorders also have an increased risk for these conditions.
- Findings of the study support the notion that eating disorders and neurodevelopmental conditions share familial risk factors, that is, genetic susceptibility and shared environmental influences.
- When treating eating disorders, it is important to recognize that some individuals have co‐occurring neurodevelopmental conditions. Adapting treatment to accommodate their needs may help improve both the quality of care and treatment outcomes.

## 1 Introduction

Eating disorders (EDs), such as anorexia nervosa (AN), bulimia nervosa (BN), and other EDs, are all characterized by dysregulated eating behavior. Previous register‐based studies have suggested that individuals with AN are approximately eight times more likely to have autism spectrum disorder (ASD), three times more likely to have attention*‐*deficit/hyperactivity disorder (ADHD), and ten times more likely to have obsessive‐compulsive disorder (OCD) (Cederlöf et al. 2015; Christiansen et al. 2024; Koch et al. 2015). To our knowledge, no previous register‐based studies have assessed the prevalence of tic disorders (TD), including Tourette syndrome, among individuals with EDs. Co‐occurring neurodevelopmental conditions have been shown to be particularly overrepresented among those who experience serious life‐threatening complications from EDs, need a long hospital stay with intensive treatment, have EDs with severe clinical features, and experience a poor treatment outcome (Kemp et al. 2023; Keski‐Rahkonen and Ruusunen 2023; Nazar et al. 2018; Nielsen et al. 2022; Zhang et al. 2022). This suggests that neurodivergence may contribute to both the emergence and persistence of complex ED presentations.

Growing evidence supports the presence of shared genetic, neurodevelopmental, and neurocognitive characteristics between neurodevelopmental conditions and EDs (Anttila et al. 2018; Cederlöf et al. 2015; Christiansen et al. 2024; Lee et al. 2019). Register‐based studies demonstrate bidirectional associations between AN and ASD, as well as elevated familial co‐aggregation, supporting shared liability across generations (Christiansen et al. 2024; Koch et al. 2015; Steinhausen et al. 2021). Neurocognitive characteristics commonly associated with ASD, such as cognitive rigidity, detail‐focused processing, and social‐cognitive difficulties, are frequently observed in individuals with EDs and in unaffected relatives of those with AN, indicating a familial aggregation of ASD‐related cognitive traits (Fuglset 2019; Galimberti et al. 2013; Tapajóz et al. 2019). EDs are also consistently associated with ADHD. Population‐based studies report bidirectional associations between ADHD and multiple ED phenotypes, potentially reflecting shared underlying mechanisms related to impulsivity, emotion regulation, and reward processing (Christiansen et al. 2024; Yao et al. 2021). In addition, EDs and OCD share overlapping neurocognitive features, including perfectionism and a tendency toward rigid behavior (Touzeau et al. 2025; Vanzhula et al. 2021). Bidirectional associations between AN and OCD have been demonstrated in a register study, along with elevated occurrence of OCD among unaffected relatives of individuals with AN, suggesting shared familial and genetic vulnerability (Cederlöf et al. 2015; Hofer et al. 2018). Evidence for associations between EDs and TD is more limited, though selective and restrictive eating behaviors have been reported in individuals with Tourette syndrome (Smith et al. 2019, 2022).

Genetic studies support shared etiology between EDs and neurodevelopmental conditions. Moderate genetic correlations and shared polygenic risk scores have been reported between AN and both ASD and OCD, whereas ADHD shows stronger genetic links with binge eating, BN, and higher body mass index (Anttila et al. 2018; Grotzinger et al. 2025; Hübel et al. 2021; Meijsen et al. 2025; Schork et al. 2019; Yao et al. 2021). These overlaps may be partly explained by pleiotropy, whereby the same genetic variants contribute to liability for multiple disorders (Lee et al. 2019). There is limited research of a possible genetic relationship between TDs and EDs; these associations have been nonsignificant in genome‐wide association studies (Anttila et al. 2018; Lee et al. 2019). In general, genetic associations and familial aggregation observed across neuropsychiatric and psychiatric disorders suggest that shared genetic vulnerability is not specific to EDs and neurodevelopmental conditions, but rather reflects a more general phenomenon spanning multiple psychiatric disorders (Anttila et al. 2018; Lee et al. 2019).

This study aimed to investigate the occurrence of ASD, ADHD, TD, and OCD in individuals with different EDs (AN, BN, other/unspecified EDs) compared to matched population controls. To examine potential familial aggregation of neurodevelopmental conditions and OCD with EDs, we investigated the occurrence of these conditions among full and half siblings of individuals with EDs compared to the full and half siblings of the matched population controls.

## 2 Method

### 2.1 Study Population

Our study population included all individuals born in Finland between January 1, 1991, and December 31, 2001, who had a primary or a secondary diagnosis in the inpatient or outpatient records of ICD‐10 ED diagnosis between 1998 and 2011. In addition, full and half siblings of ED participants and full and half siblings of population controls were included. The individuals with any of the following ICD‐10 ED diagnoses since age six were included: AN (including F50.0 AN and F50.1 atypical AN), BN (including F50.2 BN and F50.3 atypical BN), and other/unspecified EDs (including F50.8 other EDs and F50.9 ED, unspecified). If participants had more than one recorded ED diagnosis (F50 category), the first given ED diagnosis was used.

Each index individual with an ED was matched with up to four control individuals who had no recorded lifetime ED diagnoses by the end of the follow‐up according to the following criteria: date of birth (+/−30 days), registered sex (hence referred to as sex), and place of birth. Most cases had four controls, but for some, there were less individuals fulfilling all matching criteria available. In these cases, two or three controls are included. We further identified all full and half siblings of participants with EDs and of the control participants. All participants were followed up since the date of birth to death, emigration, or the end of year 2011, whichever came first.

### 2.2 Neurodevelopmental Disorders and OCD

Neurodevelopmental diagnoses were retrieved from primary and secondary diagnoses in the inpatient and outpatient records of the Finnish Care Register for Health Care. The following four neurodevelopmental diagnostic categories were included: ASD (ICD‐10 codes F84.0, F84.1, F84.5, F84.8, F84.9), ADHD (ICD‐10 codes F90.0. F90.1, F90.8, F90.9, and F98.8, used for inattentive subtype of ADHD in Finland), TD including Tourette syndrome (ICD‐10 codes F95.0, F95.1, F95.2, F95.8, F95.9), and OCD (ICD‐10 codes F42.0, F42.1, F42.2, F42.8, F42.9). The co‐occurring neurodevelopmental conditions were diagnosed either before or after the first ED diagnosis. In Finland, neurodevelopmental diagnoses are assigned by attending physicians following a comprehensive diagnostic evaluation, primarily in the public health care, or—to a lesser extent—in the private health care. Finland has a comprehensive public healthcare system, and public providers of specialized healthcare are required to report all registered diagnoses to the national registers described below.

### 2.3 Study Registers

The study capitalized on the use of the following Finnish registries: Central Population Register, the Medical Birth Register, and the Finnish Care Register for Health Care. All individuals in these registries can be identified by a unique personal identification number assigned at birth or migration, which allows for linkages between each of the registries.

### 2.4 The Central Population Register

The register includes comprehensive data on place of birth, date of emigration, date of death, place of residence, and biological parents, including their birth dates. The register is maintained by the Digital and Population Data Services Agency.

### 2.5 The Medical Birth Register

The register is a national birth register instituted by the Finnish Institute for Health and Welfare since 1987. It contains information on all births in Finland.

### 2.6 The Care Register for Health Care

As a continuation the Finnish Hospital Discharge Register, the register identifies all recorded diagnoses for all hospital admissions, including psychiatric hospitals, and recorded diagnoses for all specialized outpatient care. All Finnish citizens and permanent residents are entitled to Finland's national health insurance maintained by the state and financed through tax revenues. The registries contain the primary diagnoses together with possible subsidiary diagnoses. Diagnostic information is based on clinical diagnoses made by the attending physician. The register is maintained by the Finnish Institute for Health and Welfare.

### 2.7 Ethical Permission

Ethical permits for the study were obtained from the hospital district of Southwest Finland Ethics Committee (record number 110/180/2008, date October 21st, 2008), and the Finnish Institute for Health and Welfare (record number 463/5.05.00/2019, date May 15th, 2020). Because the data were completely register‐based, personal informed consent was not required.

### 2.8 Statistical Analysis

Appropriate to the matched case–control study design, conditional logistic regression was used for obtaining odds ratios (ORs) with 95% confidence intervals (CIs) for neurodevelopmental conditions and OCD. The first analysis included all EDs in the same analysis. Next, the following ED categories were analyzed separately: AN (F50.0, F50.1), BN (F50.2, F50.3), and other/unspecified EDs (F50.8, F50.9). ASD, ADHD, TD, and OCD were included separately in all analyses. Following the main analysis, we analyzed females and males separately. We also conducted supplementary analyses in which each included ED diagnostic code (F50.0, F50.1, F50.2, F50.3, F50.8, F50.9) was examined separately.

In the sibling analyses, we estimated ORs with 95% CIs for neurodevelopmental conditions among full siblings and half siblings of individuals with ED (exposure) compared to full and half siblings of control individuals. Sibling analyses were adjusted for date of birth, region of birth, and sex. Siblings were classified according to the pooled diagnostic category defined by the index individual's ED diagnosis, consistent with the approach used for index individuals. ASD, ADHD, TD, and OCD were analyzed separately throughout. After the main analysis, we analyzed females and males separately.

Statistical significance was defined as *p* < 0.0125 following Bonferroni correction for four different outcomes (ASD, ADHD, TD, and OCD; 0.05/4). Statistical analyses were performed with Stata, version 19.5. ChatGPT was used to support statistical coding by generating specific code segments and debugging code. All codes were reviewed, approved, and statistical analyses were conducted by the authors, who take full responsibility for the manuscript.

## 3 Results

### 3.1 Participants

The study population comprised 4240 individuals with ED and 16,494 control individuals. In sibling analyses, there were 5214 full siblings of participants with ED and 23,641 full siblings of control participants. Further, a total of 1555 half siblings of ED participants and 5891 half siblings of control participants were included. 75.0% of individuals with ED and 78.2% of control individuals had at least one sibling. See Table S1 for number of siblings in individuals with ED and control individuals. Among individuals diagnosed with ED, 90.0% were females and 10.0% were males. Mean age at ED diagnosis was 14.94 years (standard deviation 2.42). See Table 1 for participant characteristics and Table S2 for concordant neurodevelopmental diagnoses between index individuals and siblings. Ethnic background of the participants is not reported because it is not available in the register data.

**TABLE 1:** Participant characteristics. Control and sibling participants were categorized according to the ED subtype of their corresponding index individual and are therefore presented within pooled ED categories.

		Participants with ED	Control participants	Full siblings of ED participants	Full siblings of control participants	Half siblings of ED participants	Half siblings of control participants
Total *N*		4240	16,494	5214	23,641	1555	5891
Registered sex	Females *N* (%)	3814 (90.0%)	14,828 (90.0%)	2599 (49.8%)	11,487 (48.6%)	774 (49.8%)	2893 (49.1%)
	Males *N* (%)	426 (10.0%)	1666 (10.0%)	2615 (50.2%)	12,154 (51.4%)	781 (50.2%)	2998 (50.9%)
ED diagnoses/pooled ED category of the control and sibling participants	Anorexia nervosa *N* (%)	2118 (50.0%)	8233 (50.0%)	2553 (49.0%)	11,319 (47.9%)	643 (41.4%)	2864 (48.6%)
	Bulimia nervosa *N* (%)	509 (12.0%)	1990 (12.0%)	570 (10.9%)	2631 (11.1%)	195 (12.5%)	709 (12.0%)
	Other/unspecified ED *N* (%)	1613 (38.0%)	6271 (38.0%)	2091 (40.1%)	9691 (41.0%)	717 (46.1%)	2318 (39.3%)
Neurodevelopmental diagnoses	ASD *N* (%)	109 (2.6%)	58 (0.4%)	75 (1.5%)	164 (0.7%)	11 (0.7%)	35 (0.6%)
	ASD in females *N* (%)	66 (1.7%)	51 (0.3%)	18 (0.7%)	33 (0.3%)	N/A	5 (0.2%)
	ASD in males *N* (%)	43 (10.1%)	7 (0.4%)	57 (2.2%)	131 (1.1%)	N/A	30 (1.0%)
	ADHD *N* (%)	117 (2.8%)	161 (1.0%)	103 (2.0%)	305 (1.3%)	34 (2.2%)	121 (2.1%)
	ADHD in females *N* (%)	82 (2.2%)	116 (0.8%)	33 (1.3%)	42 (0.7%)	10 (1.3%)	21 (0.7%)
	ADHD in males *N* (%)	35 (8.2%)	45 (2.7%)	70 (2.7%)	263 (2.2%)	24 (3.1%)	100 (3.3%)
	TD *N* (%)	22 (0.5%)	35 (0.2%)	26 (0.5%)	76 (0.3%)	5 (0.3%)	18 (0.3%)
	TD in females *N* (%)	13 (0.3%)	29 (0.2%)	N/A	20 (0.2%)	N/A	5 (0.2%)
	TD in males *N* (%)	9 (2.1%)	6 (0.4%)	N/A	56 (0.5%)	N/A	13 (0.4%)
	OCD *N* (%)	157 (3.7%)	76 (0.5%)	30 (0.6%)	48 (0.2%)	< 5 (< 0.3%)	11 (0.2%)
	OCD in females *N* (%)	137 (3.6%)	70 (0.5%)	17 (0.7%)	25 (0.2%)	N/A	N/A
	OCD in males *N* (%)	20 (4.7%)	6 (0.4%)	13 (0.5%)	23 (0.2%)	N/A	N/A
Mother's socioeconomic status	Upper white‐collar *N* (%)	775 (18.3%)	2434 (14.8%)	766 (14.7%)	2765 (11.7%)	167 (10.7%)	531 (9.0%)
	Lower white‐collar *N* (%)	1853 (43.7%)	7529 (45.6%)	1808 (34.7%)	8338 (35.3%)	421 (27.1%)	1802 (30.6%)
	Blue collar worker *N* (%)	650 (15.3%)	2982 (18.1%)	522 (10.0%)	2867 (12.1%)	273 (17.6%)	1026 (17.4%)
	Other *N* (%)	648 (15.3%)	2402 (14.6%)	732 (14.0%)	3641 (15.4%)	236 (15.2%)	826 (14.0%)
	Missing information *N* (%)	314 (7.4%)	1147 (6.9%)	1386 (26.6%)	6030 (25.5%)	458 (29.4%)	1706 (29.0%)

*Note:* In sex‐stratified tables, when a category contained more than 0 but fewer than 5 individuals, that category and the corresponding category for the other sex were marked as not applicable (N/A) to protect individual privacy.

Abbreviations: ADHD, attention‐deficit/hyperactivity disorder; ASD, autism spectrum disorder: ASD; ED, eating disorder; OCD, obsessive‐compulsive disorder; TD, tic disorder.

### 3.2 ASD In Individuals With EDs and Their Siblings

In total, 109 (2.6%) of the 4240 individuals with EDs and 58 (0.4%) of the 16,494 population controls were diagnosed with ASD (Figure 1, Figure S1 and Table S3). Individuals with AN and other/unspecified EDs had an elevated occurrence for ASD (OR 6.62 [95% CI 4.21–10.39]; OR 9.06 [95% CI 5.55–14.77], respectively). In the sex‐stratified analyses, the occurrence of ASD was particularly high among males with AN and with other/unspecified EDs: of the 179 males with AN, 17 (9.5%) and of the 226 males with other/unspecified EDs, 25 (11.1%) were diagnosed with ASD (OR 16.58 [95% CI 5.58–42.29]; OR 32.40 [95% CI 9.78–107.39], respectively).

**FIGURE 1:**
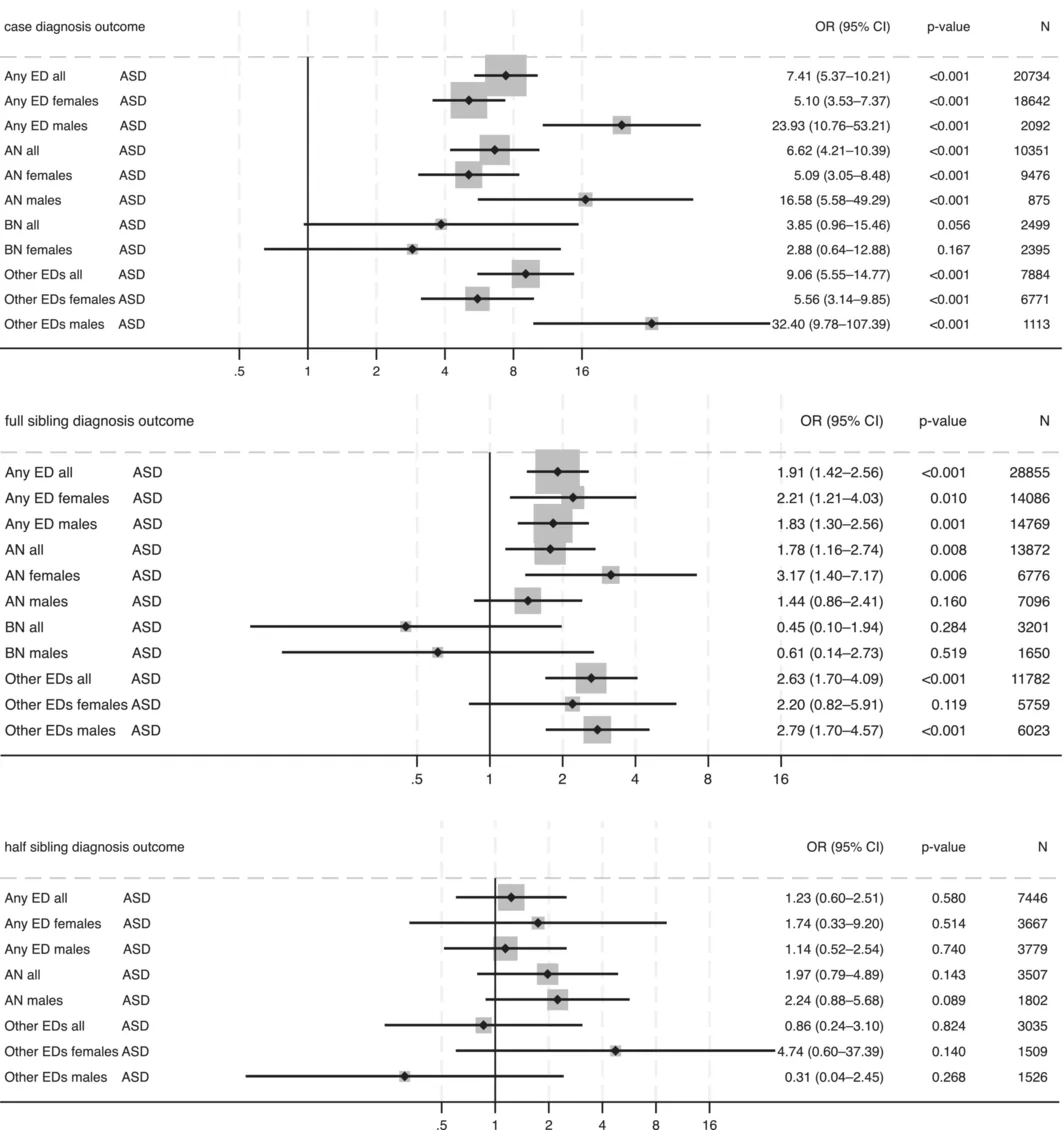
Odds ratios (ORs) and 95% confidence interval (CI) of autism spectrum disorder (ASD) in individuals with a diagnosed eating disorder (ED) and their full siblings and half siblings compared with their population controls. The horizontal line indicates the 95% confidence interval, and the size of the gray square reflects the precision of the effect estimate. AN, Anorexia nervosa; BN, bulimia nervosa.

Among the 5214 full siblings of individuals with ED, 75 (1.4%), and among the 23,641 full siblings of population controls, 164 (0.7%) were diagnosed with ASD. Full siblings of individuals with AN or other/unspecified EDs had an elevated occurrence for ASD (OR 1.78 [95% CI 1.16–2.74]; OR 2.63 [95% CI 1.70–4.09], respectively). Sex‐stratified analyses showed that the occurrence of ASD was elevated only among female full siblings of individuals with AN and among male full siblings of individuals with other/unspecified EDs (OR 3.71 [95% CI 1.40–7.17]; OR 2.79 [95% CI 1.70–4.57], respectively).

In total, 11 (0.7%) of the 1555 half siblings of individuals with ED and 35 (0.6%) of the 5891 half siblings of population controls were diagnosed with ASD. Half siblings of individuals with ED did not have an elevated occurrence of ASD.

### 3.3 ADHD In Individuals With EDs and Their Siblings

In total, 117 (2.8%) of the 4240 individuals with EDs and 161 (1.0%) of the 16,494 population controls were diagnosed with ADHD (Figure 2, Figure S2 and Table S4). Individuals with AN and other/unspecified EDs had an elevated occurrence of ADHD (OR 3.30 [95% CI 2.33–4.69]; OR 2.78 [95% CI 1.92–4.01], respectively). There were no marked sex differences in the elevated occurrence of ADHD among individuals with ED.

**FIGURE 2:**
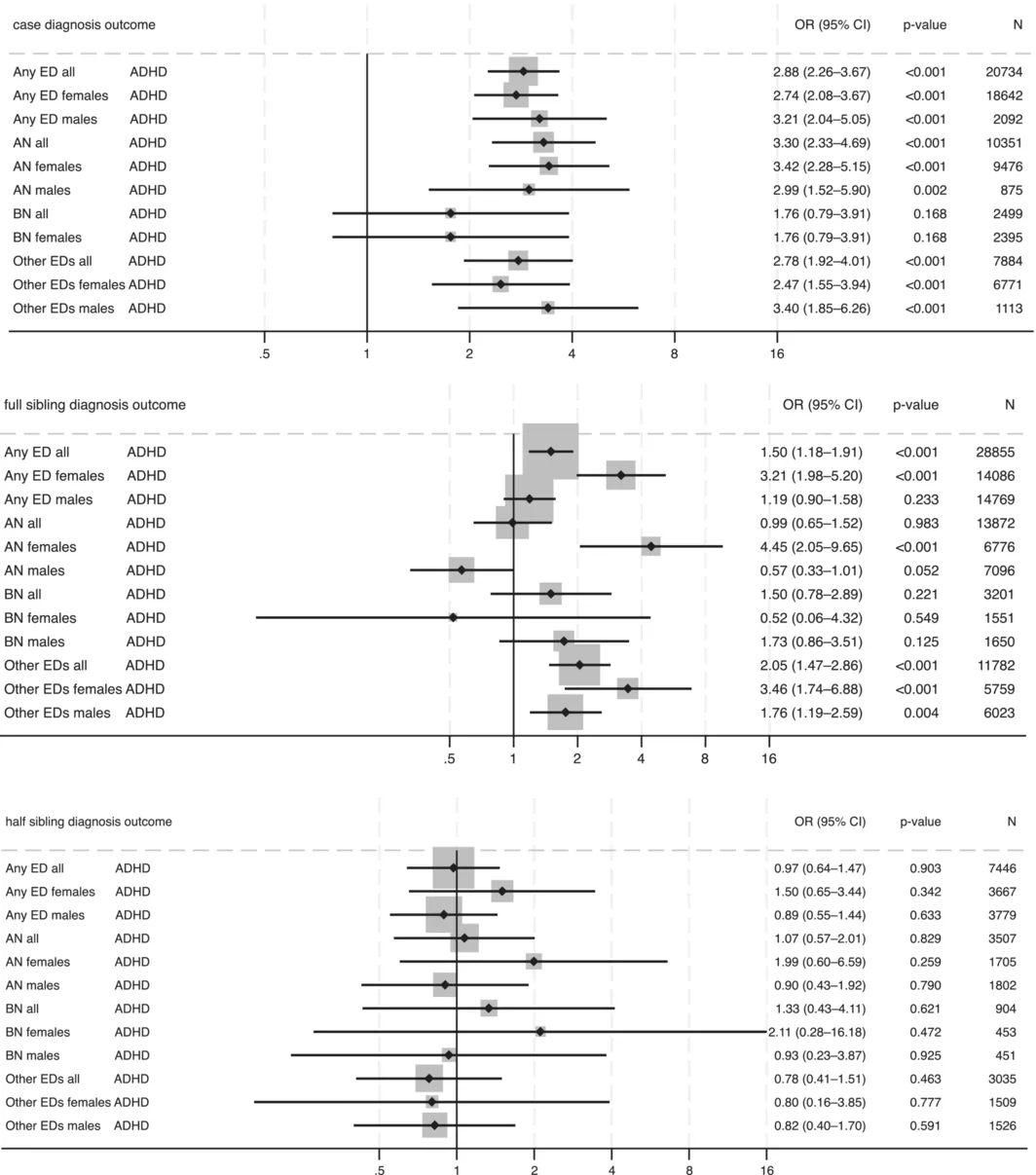
Odds ratios (ORs) and 95% confidence interval (CI) of attention‐deficit/hyperactivity disorder (ADHD) in individuals with a diagnosed eating disorder (ED) and their full siblings and half siblings compared with their population controls. The horizontal line indicates the 95% confidence interval, and the size of the gray square reflects the precision of the effect estimate. AN, Anorexia nervosa; BN, bulimia nervosa.

Among the 5214 full siblings of individuals with ED, 103 (2.0%), and among the 23,641 full siblings of population controls, 305 (1.3%) were diagnosed with ADHD. Female full siblings of individuals with AN had increased occurrence of ADHD (OR 4.45 [95% CI 2.05–9.65]). Full siblings of individuals with other/unspecified ED had an elevated occurrence for ADHD (OR 2.05 [95% CI 1.47–2.86]), and this was observed in sex‐stratified analyses in both females and males (OR 3.46 [95% CI 1.74–6.88]; OR 1.76 [95% CI 1.19–2.59], respectively).

In total, 34 (2.2%) of the 1555 half siblings of individuals with ED and 121 (2.1%) of the 5891 half siblings of population controls were diagnosed with ADHD. Half siblings of individuals with ED did not have an elevated occurrence of ADHD.

### 3.4 TD In Individuals With EDs and Their Siblings

In total, 22 (0.5%) of the 4240 individuals with EDs and 35 (0.2%) of the 16,494 population controls were diagnosed with TD (Figure 3, Figure S3 and Table S5). Individuals with other/unspecified ED had an elevated occurrence for TD when females and males were analyzed together (OR 4.68 [95% CI 1.94–11.32]). However, in sex‐stratified analyses, only males with other/unspecified EDs showed a significantly elevated occurrence of TD (OR 7.00 [95% CI 2.05–23.91]).

**FIGURE 3:**
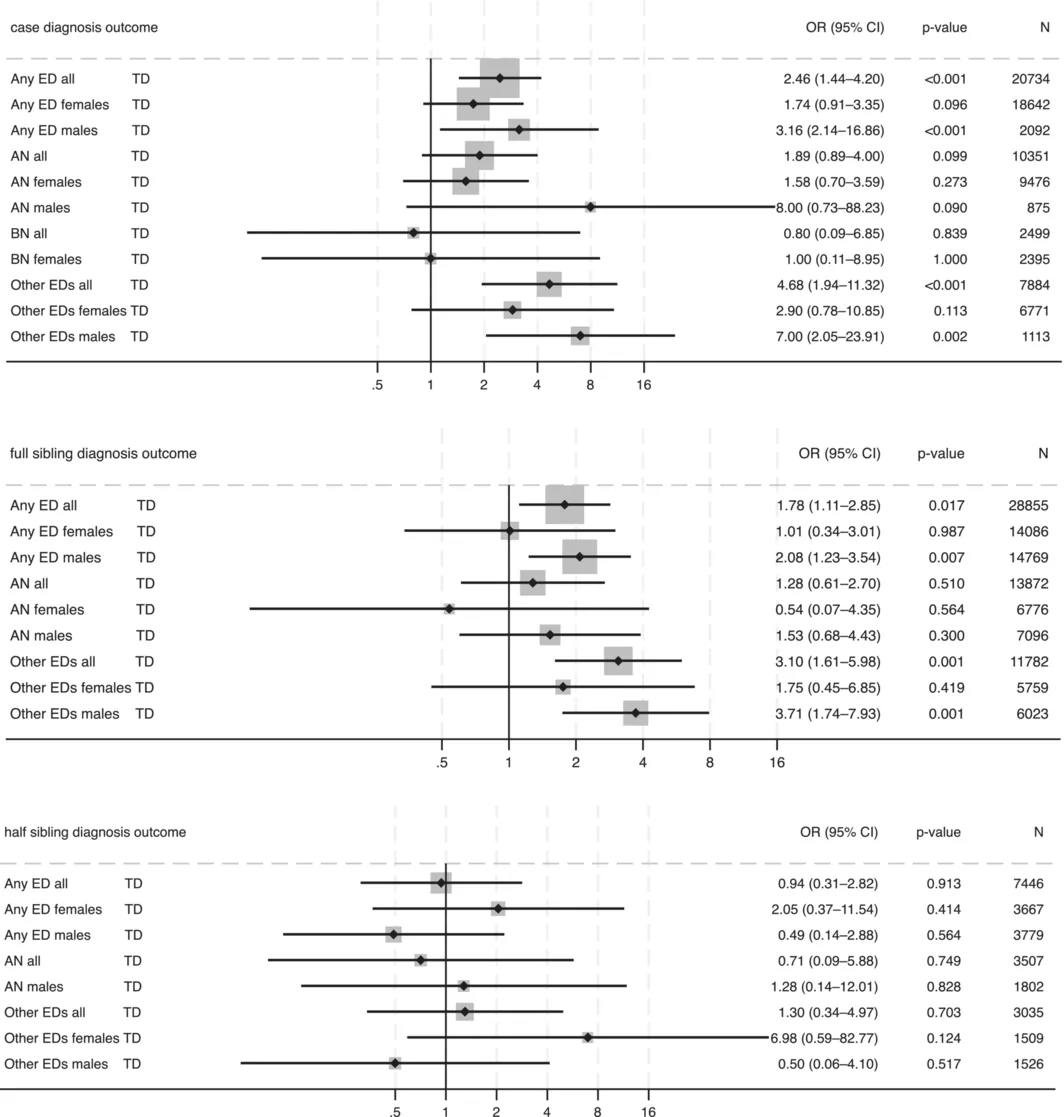
Odds ratios (ORs) and 95% confidence interval (CI) of tic disorders (TD) in individuals with a diagnosed eating disorder (ED) and their full siblings and half siblings compared with their population controls. The horizontal line indicates the 95% confidence interval, and the size of the gray square reflects the precision of the effect estimate. AN, Anorexia nervosa; BN, bulimia nervosa.

Among the full siblings of individuals with ED, 26 (0.5%) and among the full siblings of population controls 76 (0.3%) were diagnosed with TD. Full siblings of individuals with other/unspecified EDs had an elevated occurrence for TD (OR 3.10 [95% CI 1.61–5.98]). However, in the sex‐stratified analyses, only male full siblings of individuals with other/unspecified EDs exhibited an elevated occurrence of TD, mirroring the pattern observed among index individuals with ED (OR 3.71 [95% CI 1.74–7.93]).

In total, 18 (0.3%) of the 1555 half siblings of individuals with ED and 5 (0.3%) of the 5891 half siblings of population controls were diagnosed with TD. Half siblings of individuals with ED did not have an elevated occurrence for TD.

### 3.5 OCD In Individuals With EDs and Their Siblings

In total, 157 (3.7%) of the 4240 individuals with ED and 76 (0.5%) of the 16,494 population controls were diagnosed with OCD (Figure 4, Figure S4 and Table S6). Individuals with AN, BN, and other/unspecified EDs had an elevated occurrence for OCD (OR 10.67 [95% CI 7.07–16.10]; OR 3.39 [95% CI 1.57–7.33]; OR 7.64 [95% CI 4.92–11.87], respectively). There were no marked sex differences in the elevated occurrence of OCD among individuals with ED.

**FIGURE 4:**
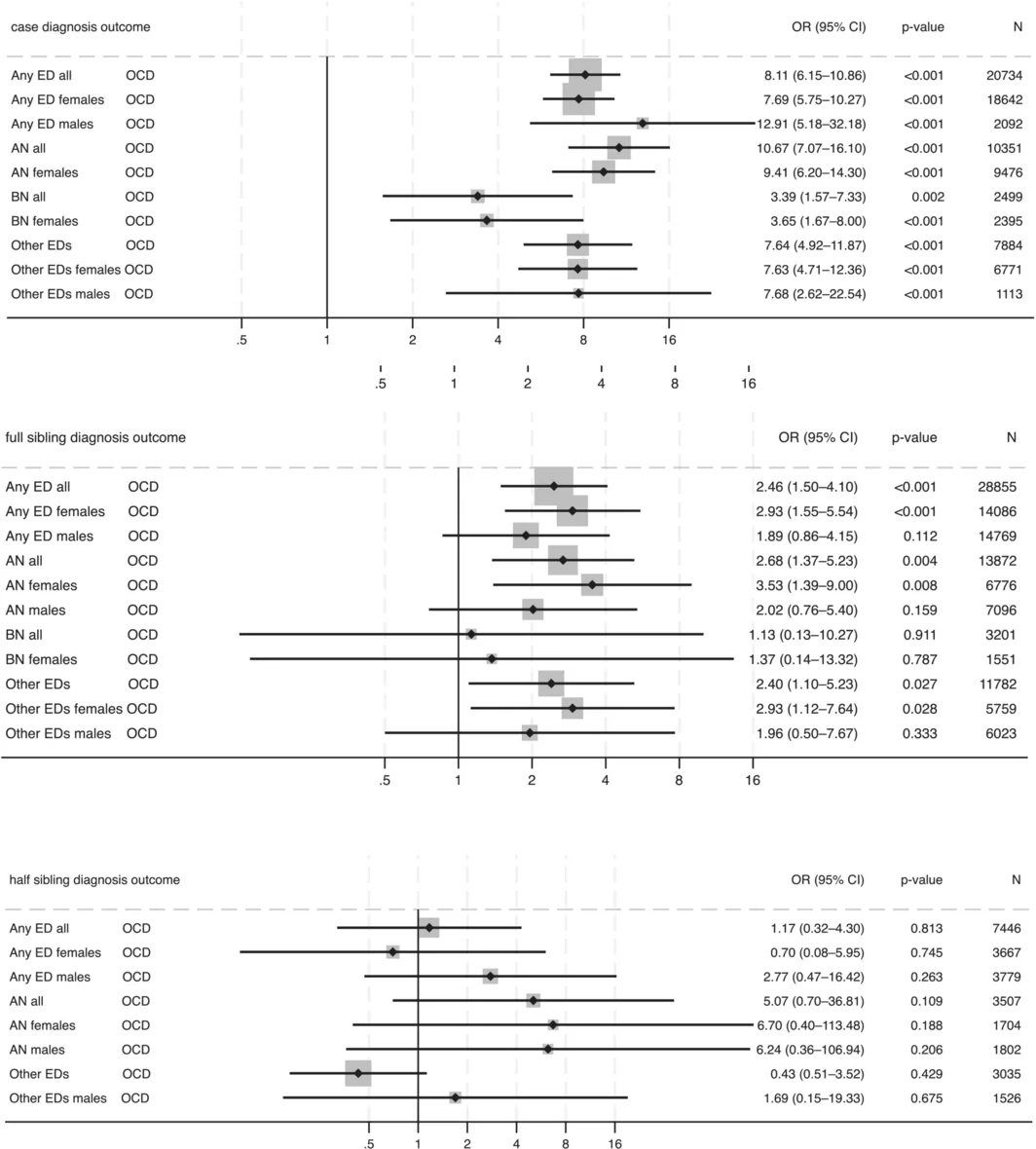
Odds ratios (ORs) and 95% confidence interval (CI) of obsessive‐compulsive disorder (OCD) in individuals with a diagnosed eating disorder (ED) and their full siblings and half siblings compared with their population controls. The horizontal line indicates the 95% confidence interval, and the size of the gray square reflects the precision of the effect estimate. AN, Anorexia nervosa; BN, bulimia nervosa.

Among the 5214 full siblings of individuals with ED, 30 (0.6%), and among the 23,641 full siblings of population controls, 48 (0.2%) were diagnosed with OCD. Full siblings of individuals with AN had an elevated occurrence for OCD (OR 2.68 [95% CI 1.37–5.23]), but in sex‐stratified analyses, only female full siblings of individuals with AN showed significantly elevated occurrence of OCD (OR 3.53 [95% CI 1.39–9.00]).

In total, less than 5 (< 0.3%) of the 1555 half sibling of individuals with ED and 11 (0.2%) of the 5891 half siblings of population controls were diagnosed with OCD. Half siblings of individuals with EDs did not have an elevated occurrence of OCD.

## 4 Discussion

In this study, we found that individuals with EDs had strikingly elevated occurrences of ASD, ADHD, TD, and OCD. The co‐occurrence of ASD was found to be particularly high among males: 9.5% of males with AN and 11.1% of males with other/unspecified EDs had ASD diagnosis; among their population controls, these proportions were 0.7% and 0.6%, respectively. Full siblings of individuals with AN or other/unspecified ED also had an elevated occurrence of ASD, ADHD, TD, and OCD, though the increase was less pronounced compared with that in index individuals. Half siblings of individuals with ED showed no elevated occurrence of studied conditions. The observed gradient across degrees of relatedness is consistent with the contribution of genetic and shared environmental factors to the familial liability underlying the co‐occurrence of EDs and neurodevelopmental conditions.

We found the occurrence of ASD, ADHD and OCD to be elevated in individuals with AN. Our findings of a six‐fold increased odds for ASD and a three‐fold increased odds for ADHD among individuals with AN are broadly consistent with a previous Danish register‐based study, which reported an approximately eight‐fold higher hazard of ASD and a three‐fold higher hazard of ADHD in individuals with AN compared to population controls (Christiansen et al. 2024). Our findings regarding the 10‐fold elevated odds for OCD in individuals with AN are also consistent with a previous register‐based study, likewise reporting 10‐fold increased risk ratio of OCD in individuals with prior AN (Cederlöf et al. 2015). Furthermore, we also found elevated occurrence of ASD, ADHD, TD, and OCD in individuals with other/unspecified EDs. We observed a nine‐fold increased odds for ASD and a three‐fold increased odds for ADHD among individuals with other/unspecified EDs, which are consistent with a previous register‐based study demonstrating approximately eight‐fold and four‐fold higher hazard for ASD and ADHD, respectively (Christiansen et al. 2024). To our knowledge, this is the first register‐based study to report an elevated occurrence of TD in individuals with EDs, providing novel evidence of an increased likelihood of TD in individuals with other/unspecified EDs.

The other/unspecified EDs category includes types of EDs that do not fulfill AN or BN criteria, are atypical, or are not included in the ICD‐10 classification as specific diagnoses such as avoidant/restrictive food intake disorder (ARFID). ARFID is characterized by restrictive eating without body image disturbances, rather motivated, for instance, by sensory processing sensitivity. ARFID frequently co‐occurs with neurodevelopmental conditions, particularly ASD and TD (Koomar et al. 2021; Nyholmer et al. 2025). The absence of an ICD‐10 diagnostic code for ARFID limited our ability to identify such cases in the registry data, but it is possible that severe ARFID presentations are classified under AN or other/unspecified EDs categories. Thus, our results may partly reflect the co‐occurrence of ARFID and neurodevelopmental conditions.

Another dimension of eating pathology potentially included in other/unspecified EDs category is binge eating behavior. As ICD‐10 does not contain a specific code for binge‐eating disorder (BED), the associations observed between other/unspecified EDs and neurodevelopmental conditions may partly reflect BED‐like presentations. Previous evidence suggests that ADHD is associated with obesity (Chen et al. 2018; Waring and Lapane 2008), and with EDs characterized by the presence of binge eating, purging, and loss of control eating (Curtin et al. 2013; Villa et al. 2023). Specifically, impulsivity is associated with higher ED symptoms in youth with ADHD (Curtin et al. 2013). Genetic associations of the risk of ADHD with binge eating, higher body mass index as well as BN and other EDs have also been reported (Anttila et al. 2018; Hübel et al. 2021; Yao et al. 2019).

In sex‐stratified analyses, males with other/unspecified EDs showed 32‐fold higher odds for ASD and seven‐fold higher odds for TD compared with controls. To our knowledge, our findings are the first to indicate such an elevated likelihood of ASD and TD among males with other/unspecified EDs. The clinical presentation of EDs in males often differs from that observed in females and male ED symptomatology may align less consistently with the diagnostic criteria for narrowly defined EDs such as AN and BN (Gorrell and Murray 2019). In males, ED pathology is more often characterized by muscularity, leanness, or performance‐oriented concerns and binge‐eating symptomatology rather than by drive for thinness (Gorrell and Murray 2019; Nagata et al. 2020; Raevuori et al. 2014). In addition, both ASD and TD are more prevalent in males than females (Idring et al. 2015), and the neurocognitive traits associated with these conditions may also contribute to atypical ED presentations. Together, these factors may partly explain the observed high co‐occurrence of ASD and TD with other/unspecified EDs in males.

Underdetection of ASD in females (Fusar‐Poli et al. 2022; Gesi et al. 2021) may also underlie the sex differences in the occurrence of ASD in individuals with EDs. In previous registry‐based studies, the prevalence of ASD among individuals with EDs was reported as 1.3%–4.2% (Koch et al. 2015; Zhang et al. 2022), which is consistent with our finding of 2.8% prevalence of ASD among individuals with EDs. Studies that have specifically screened and assessed ASD in individuals with EDs have found significantly higher prevalence estimates between 20%–30% (Huke et al. 2013; Inal‐Kaleli et al. 2024). In the present study, we only had access to registered clinical diagnoses, thereby missing individuals with undiagnosed ASD, known to be more common among females than males (Idring et al. 2015). Consequently, the true co‐occurrence of EDs and ASD may be higher also among females. Another possible explanation is that studies reporting higher estimates of ASD in individuals with EDs may be capturing elevated autistic traits rather than a phenotype that meets diagnostic criteria for ASD. Because many of these studies did not include comprehensive diagnostic assessments for ASD (Inal‐Kaleli et al. 2024), this methodological issue may partly explain the discrepancy between studies relying on register‐based diagnoses and studies screening for ASD characteristics.

We found evidence of familial aggregation between EDs and ASD, ADHD, TD, and OCD. Full siblings of individuals with ED had 1.5–3‐fold elevated odds for neurodevelopmental conditions, with the odds being particularly high among the full siblings of individuals with AN and other/unspecified EDs. In contrast, among half siblings of individuals with ED, the odds for neurodevelopmental conditions aligned with that in population controls. This gradient pattern, with the strongest association in ED cases, intermediate in full siblings, and no significant association in half‐siblings, supports the presence of shared familial liability for EDs and neurodevelopmental condition strengthening the previous evidence of shared familial, including genetic background (Anttila et al. 2018; Lee et al. 2019; Yilmaz et al. 2020; Zhang et al. 2022). These findings suggest that EDs may arise from broader neurodevelopmental vulnerability, partly shared with neurodevelopmental conditions, and align with molecular genetic studies demonstrating at least moderate genetic correlations and shared polygenic risk scores between EDs and neurodevelopmental conditions (Anttila et al. 2018; Grotzinger et al. 2025; Meijsen et al. 2025). It has been suggested that pleiotropic genetic effects may underlie these associations, such that the same genetic factors may contribute to both ED psychopathology and neurodivergent characteristics (Lee et al. 2019). The co‐occurrence and familial aggregation of EDs and neurodevelopmental conditions may be thus at least partly explained by common genetic factors.

From a clinical perspective, our findings suggest that individuals with ED are more likely to be neurodivergent than the general population; this is also supported by previous studies, in which neurodivergent individuals had an increased risk for EDs (Christiansen et al. 2024; Koch et al. 2015). Since many individuals receive a neurodevelopmental diagnosis only after an ED diagnosis (Zhang et al. 2022), even though such neurodevelopmental conditions should be detectable already in childhood, it is important to systematically assess neurodevelopmental characteristics in individuals with EDs. The presentation of EDs in neurodivergent individuals may not align with traditional etiological models of EDs, which predominantly conceptualize the EDs through weight‐ and shape‐related concerns. Traditionally, ED behavior has largely been interpreted through the lens of sociocultural pressure toward thinness and body image disturbance, often overlooking additional, alternative factors contributing to disordered eating. In neurodivergent individuals, ED behavior may be better explained by neurocognitive characteristics including rigid behavior, a strong preference for predictability, routine seeking, sensory sensitivities (e.g., aversions to certain textures, tastes, or food temperatures), and difficulties with interoceptive awareness, such as accurately detecting hunger and satiety cues (Brede et al. 2020; Saure et al. 2021). Importantly, these characteristics are often clinically relevant even in individuals who do not meet full diagnostic criteria for a neurodevelopmental condition. Among neurodivergent individuals with EDs, duration of ED tends to be longer and its symptoms more severe; additionally, they do not appear to benefit enough from the current treatment methods, their treatment outcome is often poorer, and somatic complications are more common (Kemp et al. 2023; Keski‐Rahkonen and Ruusunen 2023; Zhang et al. 2022). Given the impact of neurodivergence on ED severity and treatment response, it is crucial to recognize neurodevelopmental conditions in individuals with ED and develop tailored treatment approaches to improve treatment adherence and ultimately, to enhance clinical outcomes.

### 4.1 Strengths and Limitations

A major strength of this study includes a large sample obtained using nationwide systematic sampling through national registries. We included all eligible individuals in Finland and their matched population controls, which underscores the representativeness of the findings. The inclusion of both siblings and half siblings of index and control individuals constitutes an additional strength, allowing for the investigation of familial aggregation of neurodevelopmental conditions and OCD.

Limitations include reliance on register‐based diagnostic information. Consequently, misclassification bias may have occurred, as individuals without a formal diagnosis were not captured, and subclinical symptoms of EDs and neurodevelopmental conditions could not be examined. Misclassification bias may also pertain to ED cases identified according to the first, often other/unspecified EDs diagnosis, which may subsequently evolve to a specific diagnosis (i.e., AN or BN). Future studies would benefit from taking diagnostic trajectories into account and assessing combinations of ED diagnoses in a longitudinal setting. Furthermore, we were unable to distinguish between subtypes of AN (restrictive/binge–purge AN), or between subtypes of unspecified EDs (e.g., presentations characterized by binge eating vs. avoidant/restrictive behaviors such as ARFID). In the future, following the implementation of ICD‐11, it will be possible to investigate ARFID in register‐based studies. Detection bias may arise when individuals with an existing diagnosis, already engaged in healthcare service, have higher likelihood of receiving additional comorbid diagnoses, potentially leading to an overestimation of co‐occurrence. Despite nationwide sampling, there were relatively few participants in some diagnostic categories and in male‐only analyses, limiting the assessment of associations between EDs and neurodevelopmental conditions and OCD in those categories. The youngest siblings may not yet have received diagnoses by the end of the follow‐up period, which may have led to an underestimation of the occurrence of the studied conditions among them. Further, the registers may occasionally miss diagnoses recorded only in private healthcare.

## 5 Conclusions

Our study demonstrates a robust association between EDs and ASD, ADHD, TD, and OCD, with substantially increased occurrence observed in both individuals with EDs and their full siblings, suggesting shared familial factors, including genetic liability, in this relationship. These findings underscore the need to incorporate neurodevelopmental assessments into the clinical care of individuals with EDs, which may enable earlier identification and inform more targeted, effective interventions.

## Author Contributions

**Susanna Hinkka‐Yli‐Salomäki:** resources, writing – review and editing. **Pyry N. Sipilä:** conceptualization, formal analysis, methodology, writing – review and editing. **Andre Sourander:** resources, writing – review and editing. **Emma Saure:** conceptualization, formal analysis, methodology, visualization, writing – original draft, writing – review and editing. **Elina Santti:** writing – review and editing. **Anu Raevuori:** conceptualization, methodology, project administration, resources, writing – original draft, writing – review and editing.

## Lived Experience Involvement Statement

No specific efforts were undertaken to involve persons with lived experience in the study design or execution, or in the preparation of this manuscript.

## Funding

This work was supported by the Research Council of Finland (grant 259764; AR), and the Yrjö Jahnsson Foundation (grant 20257983, ESau), the Helsinki University Hospital (grant Y221; Esau, PNS), the Finnish Medical Foundation (PNS), and the Päivikki and Sakari Sohlberg Foundation (PNS).

## Conflicts of Interest

Anu Raevuori holds equity ownership in Meru Health Inc. outside the submitted work.

## Supporting information


**Figure S1:** Odds ratios (ORs) and 95% confidence interval (CI) of autism spectrum disorder (ASD) in individuals with a diagnosed eating disorder separately in all eating disorder diagnoses and their full siblings and half siblings compared with their population controls. The horizontal line indicates the 95% confidence interval, and the size of the gray square reflects the precision of the effect estimate.

**Figure S2:** Odds ratios (ORs) and 95% confidence interval (CI) of attention‐deficit/hyperactivity disorder (ADHD) in individuals with a diagnosed eating disorder separately in all eating disorder diagnoses and their full siblings and half siblings compared with their population controls. The horizontal line indicates the 95% confidence interval, and the size of the gray square reflects the precision of the effect estimate.

**Figure S3:** Odds ratios (ORs) and 95% confidence interval (CI) of tic disorders (TD) in individuals with a diagnosed eating disorder separately in all eating disorder diagnoses and their full siblings and half siblings compared with their population controls. The horizontal line indicates the 95% confidence interval, and the size of the gray square reflects the precision of the effect estimate.

**Figure S4:** Odds ratios (ORs) and 95% confidence interval (CI) of obsessive‐compulsive disorder (OCD) in individuals with a diagnosed eating disorder separately in all eating disorder diagnoses and their full siblings and half siblings compared with their population controls. The horizontal line indicates the 95% confidence interval, and the size of the gray square reflects the precision of the effect estimate.

**Table S1:** Number of siblings in individuals with eating disorder (ED) and control individuals.
**Table S2:** Concordant neurodevelopmental diagnoses between sibling pairs.
**Table S3:** Numbers of autism spectrum disorder (ASD) diagnoses in individuals with an eating disorder (ED) and in their full siblings and half siblings and in population controls and their siblings. If there are more than 0 but fewer than 5 individuals in specific category, this is marked as < 5 to protect privacy.
**Table S4:** Numbers of attention‐deficit/hyperactivity disorder (ADHD) diagnoses in individuals with an eating disorder (ED) and in their full siblings and half siblings and in population controls and their siblings. If there are more than 0 but fewer than 5 individuals in specific category, this is marked as < 5 to protect privacy.
**Table S5:** Numbers of tic disorder (TD) diagnoses in individuals with an eating disorder (ED) and in their full siblings and half siblings and in population controls and their siblings. If there are more than 0 but fewer than 5 individuals in specific category, this is marked as < 5 to protect privacy.
**Table S6:** Numbers of obsessive‐compulsive disorder (OCD) diagnoses in individuals with an eating disorder (ED) and in their full siblings and half siblings and in population controls and their siblings. If there are more than 0 but fewer than 5 individuals in specific category, this is marked as < 5 to protect privacy.

## Data Availability

Research data are not shared.
