# Supplementary material for: Autism Spectrum Disorder, Attention‐Deficit/Hyperactivity Disorder, Tic Disorder, and Obsessive‐Compulsive Disorder in Individuals With Eating Disorders and Their Siblings: A Nationwide Finnish Cohort Study
Source: Int J Eat Disord. 2026 Jul 9;59(11):2473–85. doi: 10.1002/eat.70171 (PMC13726448; doi:10.1002/eat.70171)
Supplement: Supplementary file 1 [file EAT-59-2473-s002.pdf]

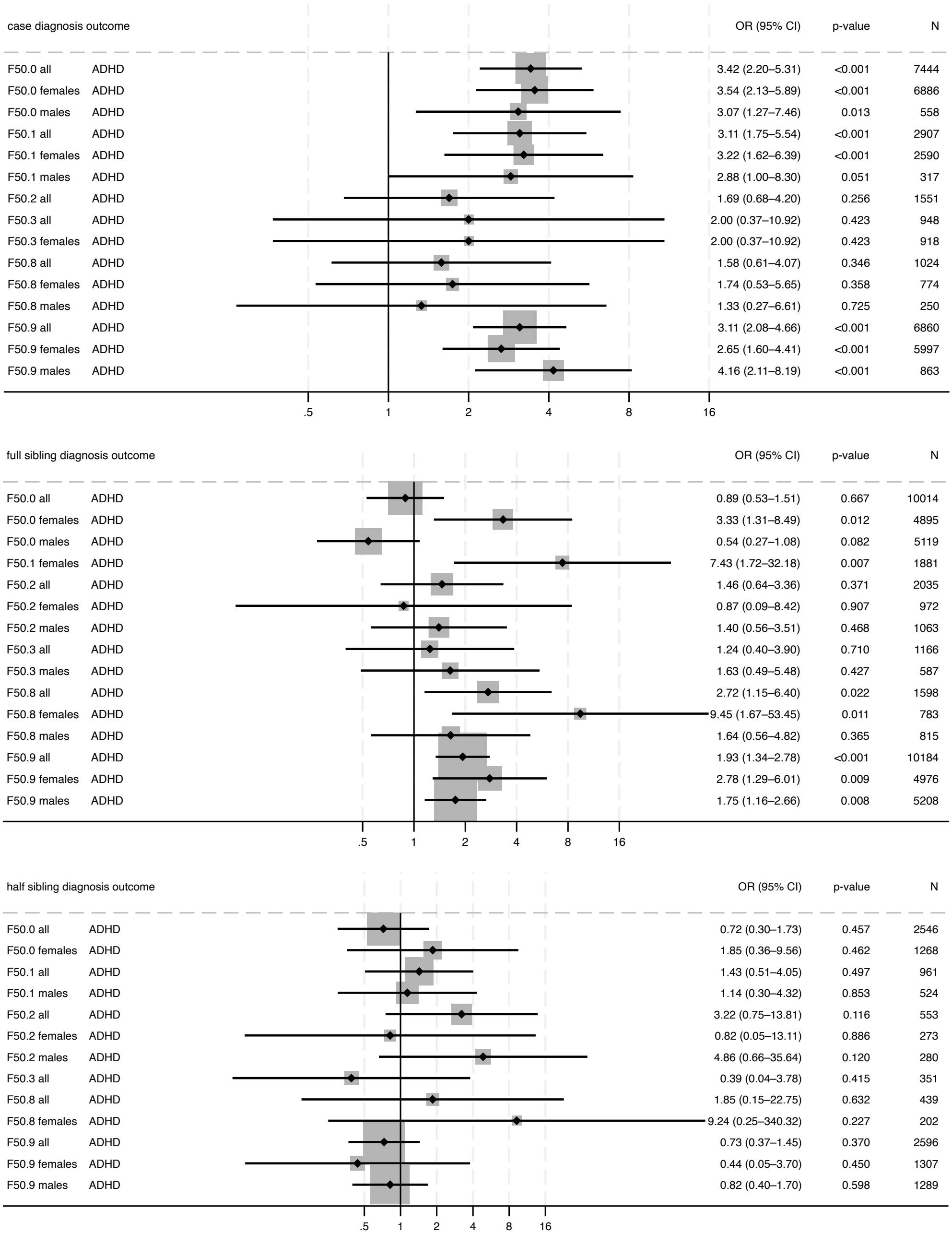
case diagnosis outcome
OR (95% CI)
p-value
N
F50.0 all ADHD 3.42 (2.20–5.31) <0.001 7444
F50.0 females ADHD 3.54 (2.13–5.89) <0.001 6886
F50.0 males ADHD 3.07 (1.27–7.46) 0.013 558
F50.1 all ADHD 3.11 (1.75–5.54) <0.001 2907
F50.1 females ADHD 3.22 (1.62–6.39) <0.001 2590
F50.1 males ADHD 2.88 (1.00–8.30) 0.051 317
F50.2 all ADHD 1.69 (0.68–4.20) 0.256 1551
F50.3 all ADHD 2.00 (0.37–10.92) 0.423 948
F50.3 females ADHD 2.00 (0.37–10.92) 0.423 918
F50.8 all ADHD 1.58 (0.61–4.07) 0.346 1024
F50.8 females ADHD 1.74 (0.53–5.65) 0.358 774
F50.8 males ADHD 1.33 (0.27–6.61) 0.725 250
F50.9 all ADHD 3.11 (2.08–4.66) <0.001 6860
F50.9 females ADHD 2.65 (1.60–4.41) <0.001 5997
F50.9 males ADHD 4.16 (2.11–8.19) <0.001 863
.5 1 2 4 8 16
full sibling diagnosis outcome
OR (95% CI)
p-value
N
F50.0 all ADHD 0.89 (0.53–1.51) 0.667 10014
F50.0 females ADHD 3.33 (1.31–8.49) 0.012 4895
F50.0 males ADHD 0.54 (0.27–1.08) 0.082 5119
F50.1 females ADHD 7.43 (1.72–32.18) 0.007 1881
F50.2 all ADHD 1.46 (0.64–3.36) 0.371 2035
F50.2 females ADHD 0.87 (0.09–8.42) 0.907 972
F50.2 males ADHD 1.40 (0.56–3.51) 0.468 1063
F50.3 all ADHD 1.24 (0.40–3.90) 0.710 1166
F50.3 males ADHD 1.63 (0.49–5.48) 0.427 587
F50.8 all ADHD 2.72 (1.15–6.40) 0.022 1598
F50.8 females ADHD 9.45 (1.67–53.45) 0.011 783
F50.8 males ADHD 1.64 (0.56–4.82) 0.365 815
F50.9 all ADHD 1.93 (1.34–2.78) <0.001 10184
F50.9 females ADHD 2.78 (1.29–6.01) 0.009 4976
F50.9 males ADHD 1.75 (1.16–2.66) 0.008 5208
.5 1 2 4 8 16
half sibling diagnosis outcome
OR (95% CI)
p-value
N
F50.0 all ADHD 0.72 (0.30–1.73) 0.457 2546
F50.0 females ADHD 1.85 (0.36–9.56) 0.462 1268
F50.1 all ADHD 1.43 (0.51–4.05) 0.497 961
F50.1 males ADHD 1.14 (0.30–4.32) 0.853 524
F50.2 all ADHD 3.22 (0.75–13.81) 0.116 553
F50.2 females ADHD 0.82 (0.05–13.11) 0.886 273
F50.2 males ADHD 4.86 (0.66–35.64) 0.120 280
F50.3 all ADHD 0.39 (0.04–3.78) 0.415 351
F50.8 all ADHD 1.85 (0.15–22.75) 0.632 439
F50.8 females ADHD 9.24 (0.25–340.32) 0.227 202
F50.9 all ADHD 0.73 (0.37–1.45) 0.370 2596
F50.9 females ADHD 0.44 (0.05–3.70) 0.450 1307
F50.9 males ADHD 0.82 (0.40–1.70) 0.598 1289
.5 1 2 4 8 16
