# Supplementary material for: Autism Spectrum Disorder, Attention‐Deficit/Hyperactivity Disorder, Tic Disorder, and Obsessive‐Compulsive Disorder in Individuals With Eating Disorders and Their Siblings: A Nationwide Finnish Cohort Study
Source: Int J Eat Disord. 2026 Jul 9;59(11):2473–85. doi: 10.1002/eat.70171 (PMC13726448; doi:10.1002/eat.70171)
Supplement: Supplementary file 2 [file EAT-59-2473-s003.pdf]

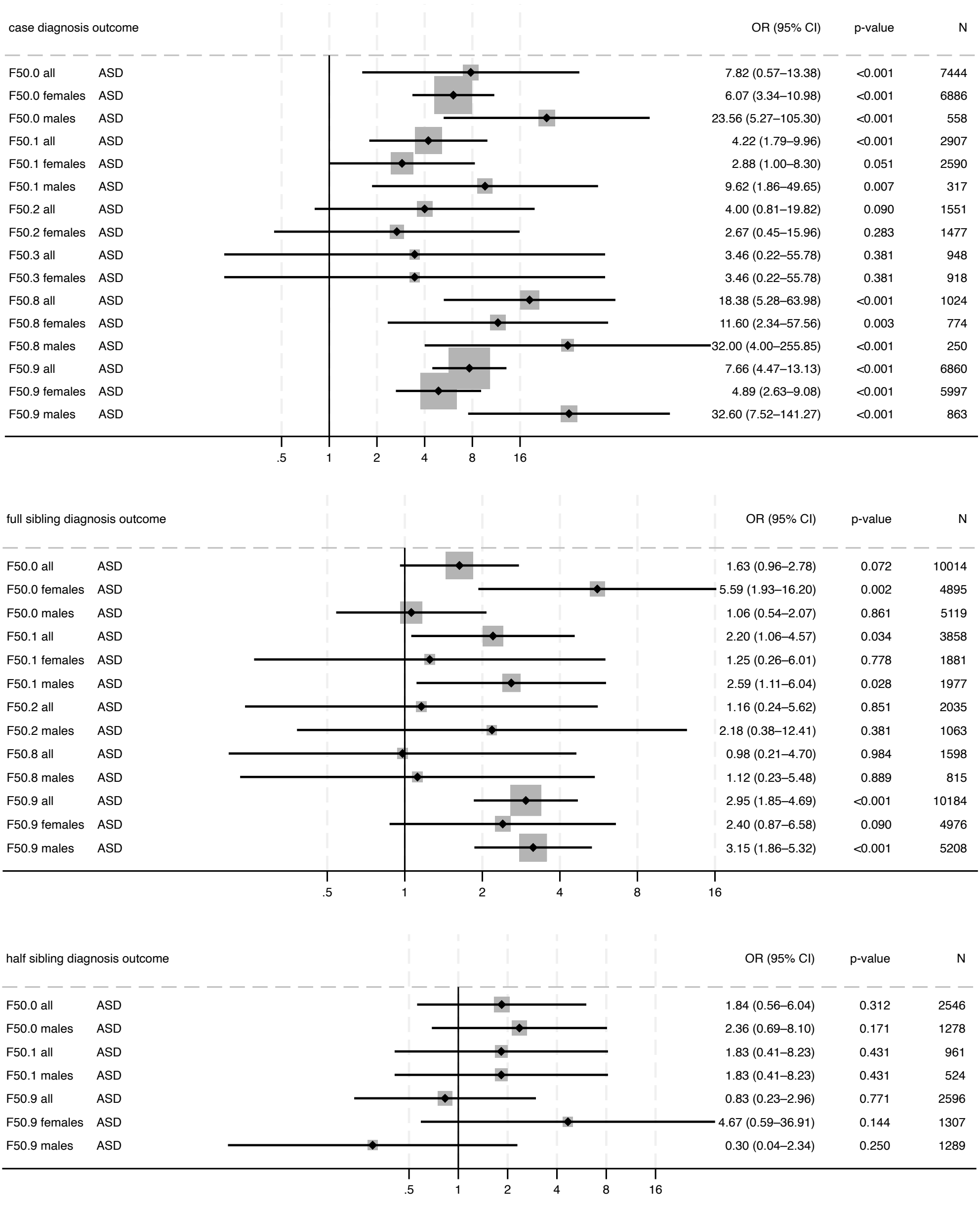

case diagnosis outcome
OR (95% CI)
p-value
N
F50.0 all ASD 7.82 (0.57–13.38) <0.001 7444
F50.0 females ASD 6.07 (3.34–10.98) <0.001 6886
F50.0 males ASD 23.56 (5.27–105.30) <0.001 558
F50.1 all ASD 4.22 (1.79–9.96) <0.001 2907
F50.1 females ASD 2.88 (1.00–8.30) 0.051 2590
F50.1 males ASD 9.62 (1.86–49.65) 0.007 317
F50.2 all ASD 4.00 (0.81–19.82) 0.090 1551
F50.2 females ASD 2.67 (0.45–15.96) 0.283 1477
F50.3 all ASD 3.46 (0.22–55.78) 0.381 948
F50.3 females ASD 3.46 (0.22–55.78) 0.381 918
F50.8 all ASD 18.38 (5.28–63.98) <0.001 1024
F50.8 females ASD 11.60 (2.34–57.56) 0.003 774
F50.8 males ASD 32.00 (4.00–255.85) <0.001 250
F50.9 all ASD 7.66 (4.47–13.13) <0.001 6860
F50.9 females ASD 4.89 (2.63–9.08) <0.001 5997
F50.9 males ASD 32.60 (7.52–141.27) <0.001 863
.5
1
2
4
8
16
full sibling diagnosis outcome
OR (95% CI)
p-value
N
F50.0 all ASD 1.63 (0.96–2.78) 0.072 10014
F50.0 females ASD 5.59 (1.93–16.20) 0.002 4895
F50.0 males ASD 1.06 (0.54–2.07) 0.861 5119
F50.1 all ASD 2.20 (1.06–4.57) 0.034 3858
F50.1 females ASD 1.25 (0.26–6.01) 0.778 1881
F50.1 males ASD 2.59 (1.11–6.04) 0.028 1977
F50.2 all ASD 1.16 (0.24–5.62) 0.851 2035
F50.2 males ASD 2.18 (0.38–12.41) 0.381 1063
F50.8 all ASD 0.98 (0.21–4.70) 0.984 1598
F50.8 males ASD 1.12 (0.23–5.48) 0.889 815
F50.9 all ASD 2.95 (1.85–4.69) <0.001 10184
F50.9 females ASD 2.40 (0.87–6.58) 0.090 4976
F50.9 males ASD 3.15 (1.86–5.32) <0.001 5208
.5
1
2
4
8
16
half sibling diagnosis outcome
OR (95% CI)
p-value
N
F50.0 all ASD 1.84 (0.56–6.04) 0.312 2546
F50.0 males ASD 2.36 (0.69–8.10) 0.171 1278
F50.1 all ASD 1.83 (0.41–8.23) 0.431 961
F50.1 males ASD 1.83 (0.41–8.23) 0.431 524
F50.9 all ASD 0.83 (0.23–2.96) 0.771 2596
F50.9 females ASD 4.67 (0.59–36.91) 0.144 1307
F50.9 males ASD 0.30 (0.04–2.34) 0.250 1289
.5
1
2
4
8
16
