# Supplementary material for: Autism Spectrum Disorder, Attention‐Deficit/Hyperactivity Disorder, Tic Disorder, and Obsessive‐Compulsive Disorder in Individuals With Eating Disorders and Their Siblings: A Nationwide Finnish Cohort Study
Source: Int J Eat Disord. 2026 Jul 9;59(11):2473–85. doi: 10.1002/eat.70171 (PMC13726448; doi:10.1002/eat.70171)
Supplement: Supplementary file 3 [file EAT-59-2473-s004.pdf]

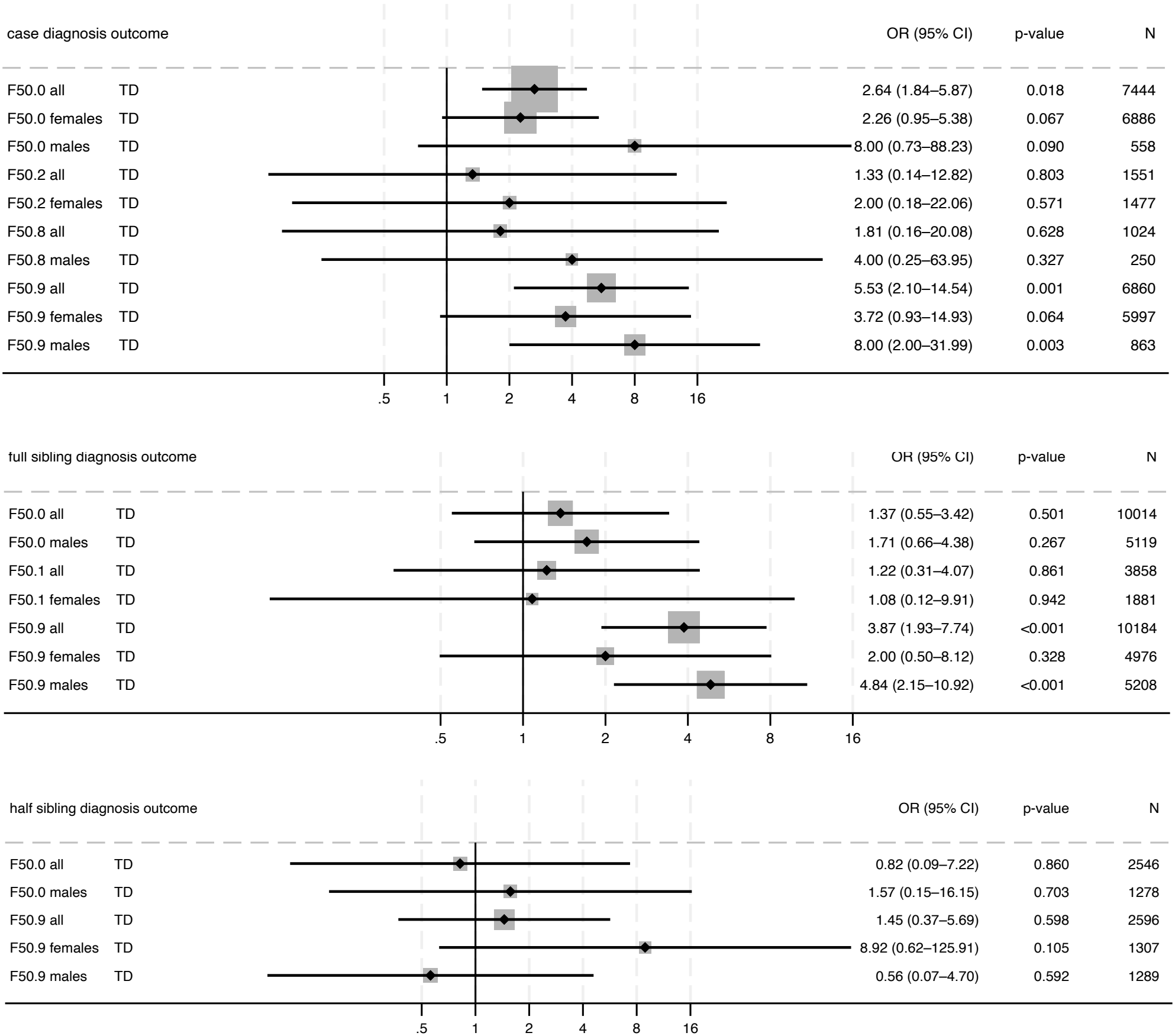
case diagnosis outcome
OR (95% CI)
p-value
N
F50.0 all TD 2.64 (1.84–5.87) 0.018 7444
F50.0 females TD 2.26 (0.95–5.38) 0.067 6886
F50.0 males TD 8.00 (0.73–88.23) 0.090 558
F50.2 all TD 1.33 (0.14–12.82) 0.803 1551
F50.2 females TD 2.00 (0.18–22.06) 0.571 1477
F50.8 all TD 1.81 (0.16–20.08) 0.628 1024
F50.8 males TD 4.00 (0.25–63.95) 0.327 250
F50.9 all TD 5.53 (2.10–14.54) 0.001 6860
F50.9 females TD 3.72 (0.93–14.93) 0.064 5997
F50.9 males TD 8.00 (2.00–31.99) 0.003 863
.5 1 2 4 8 16
full sibling diagnosis outcome
OR (95% CI)
p-value
N
F50.0 all TD 1.37 (0.55–3.42) 0.501 10014
F50.0 males TD 1.71 (0.66–4.38) 0.267 5119
F50.1 all TD 1.22 (0.31–4.07) 0.861 3858
F50.1 females TD 1.08 (0.12–9.91) 0.942 1881
F50.9 all TD 3.87 (1.93–7.74) <0.001 10184
F50.9 females TD 2.00 (0.50–8.12) 0.328 4976
F50.9 males TD 4.84 (2.15–10.92) <0.001 5208
.5 1 2 4 8 16
half sibling diagnosis outcome
OR (95% CI)
p-value
N
F50.0 all TD 0.82 (0.09–7.22) 0.860 2546
F50.0 males TD 1.57 (0.15–16.15) 0.703 1278
F50.9 all TD 1.45 (0.37–5.69) 0.598 2596
F50.9 females TD 8.92 (0.62–125.91) 0.105 1307
F50.9 males TD 0.56 (0.07–4.70) 0.592 1289
.5 1 2 4 8 16
