# Supplementary material for: Autism Spectrum Disorder, Attention‐Deficit/Hyperactivity Disorder, Tic Disorder, and Obsessive‐Compulsive Disorder in Individuals With Eating Disorders and Their Siblings: A Nationwide Finnish Cohort Study
Source: Int J Eat Disord. 2026 Jul 9;59(11):2473–85. doi: 10.1002/eat.70171 (PMC13726448; doi:10.1002/eat.70171)
Supplement: Supplementary file 4 [file EAT-59-2473-s005.pdf]

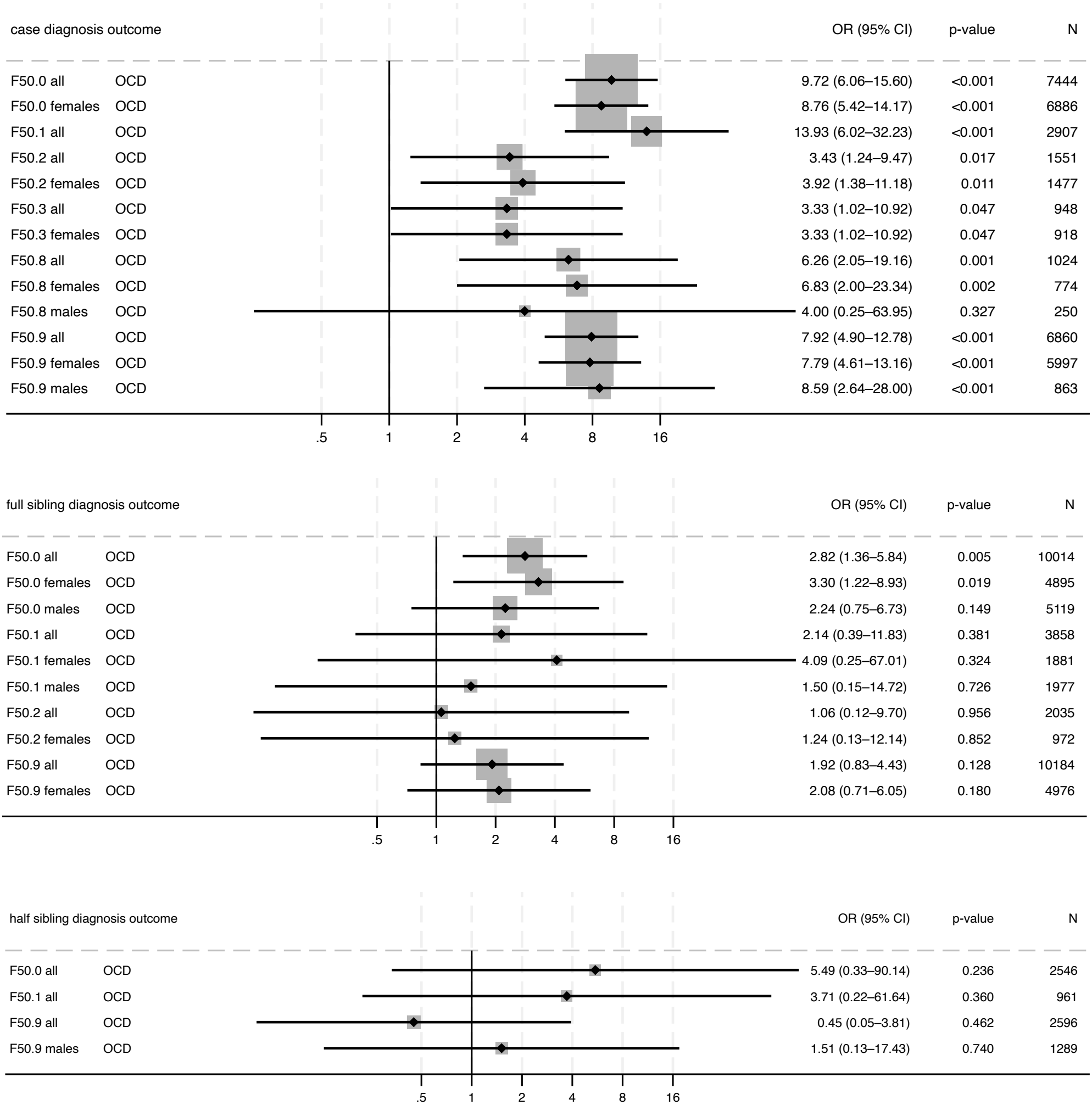
case diagnosis outcome
OR (95% CI)
p-value
N
F50.0 all OCD 9.72 (6.06–15.60) <0.001 7444
F50.0 females OCD 8.76 (5.42–14.17) <0.001 6886
F50.1 all OCD 13.93 (6.02–32.23) <0.001 2907
F50.2 all OCD 3.43 (1.24–9.47) 0.017 1551
F50.2 females OCD 3.92 (1.38–11.18) 0.011 1477
F50.3 all OCD 3.33 (1.02–10.92) 0.047 948
F50.3 females OCD 3.33 (1.02–10.92) 0.047 918
F50.8 all OCD 6.26 (2.05–19.16) 0.001 1024
F50.8 females OCD 6.83 (2.00–23.34) 0.002 774
F50.8 males OCD 4.00 (0.25–63.95) 0.327 250
F50.9 all OCD 7.92 (4.90–12.78) <0.001 6860
F50.9 females OCD 7.79 (4.61–13.16) <0.001 5997
F50.9 males OCD 8.59 (2.64–28.00) <0.001 863
.5
1
2
4
8
16
full sibling diagnosis outcome
OR (95% CI)
p-value
N
F50.0 all OCD 2.82 (1.36–5.84) 0.005 10014
F50.0 females OCD 3.30 (1.22–8.93) 0.019 4895
F50.0 males OCD 2.24 (0.75–6.73) 0.149 5119
F50.1 all OCD 2.14 (0.39–11.83) 0.381 3858
F50.1 females OCD 4.09 (0.25–67.01) 0.324 1881
F50.1 males OCD 1.50 (0.15–14.72) 0.726 1977
F50.2 all OCD 1.06 (0.12–9.70) 0.956 2035
F50.2 females OCD 1.24 (0.13–12.14) 0.852 972
F50.9 all OCD 1.92 (0.83–4.43) 0.128 10184
F50.9 females OCD 2.08 (0.71–6.05) 0.180 4976
.5
1
2
4
8
16
half sibling diagnosis outcome
OR (95% CI)
p-value
N
F50.0 all OCD 5.49 (0.33–90.14) 0.236 2546
F50.1 all OCD 3.71 (0.22–61.64) 0.360 961
F50.9 all OCD 0.45 (0.05–3.81) 0.462 2596
F50.9 males OCD 1.51 (0.13–17.43) 0.740 1289
.5
1
2
4
8
16
