# Supplementary material for: Autism Spectrum Disorder, Attention‐Deficit/Hyperactivity Disorder, Tic Disorder, and Obsessive‐Compulsive Disorder in Individuals With Eating Disorders and Their Siblings: A Nationwide Finnish Cohort Study
Source: Int J Eat Disord. 2026 Jul 9;59(11):2473–85. doi: 10.1002/eat.70171 (PMC13726448; doi:10.1002/eat.70171)
Supplement: Supplementary file 5 [file EAT-59-2473-s001.docx]

**Supplementary Table 1.** Number of siblings in individuals with eating disorder (ED) and control individuals.

| **Number of full siblings in individuals with eating disorders** | | | | | |
| --- | --- | --- | --- | --- | --- |
|  | **0**  **N (%)** | **1**  **N (%)** | **2**  **N (%)** | **3**  **N (%)** | **4 or more**  **N (%)** |
| **Any ED**  **N = 4240** | 1061  (25.0 %) | 1936  (45.7 %) | 876  (20.7 %) | 227  (5.4 %) | 140  (3.3 %) |
| **Anorexia nervosa**  **N = 2118** | 503  (23.8 %) | 999  (47.2 %) | 448  (21.2 %) | 117  (5.5%) | 51  (2.4 %) |
| **Bulimia nervosa**  **N = 509** | 149  (29.30 %) | 226  (44.4 %) | 96  (18.9 %) | 21  (4.1 %) | 17  (3.3 %) |
| **Other / unspecified ED**  **N = 1613** | 409  (25.4 %) | 711  (44.1 %) | 332  (20.6 %) | 89  (5.5 %) | 72  (4.5 %) |
| **Number of half siblings in individuals with eating disorders** | | | | | |
|  | **0**  **N (%)** | **1**  **N (%)** | **2**  **N (%)** | **3**  **N (%)** | **4 or more**  **N (%)** |
| **Any ED**  **N = 4240** | 3416  (80.6 %) | 443  (10.5 %) | 202  (4.8 %) | 102  (2.4 %) | 77  (1.8 %) |
| **Anorexia nervosa**  **N = 2118** | 1768  (83.5 %) | 183  (8.6 %) | 93  (4.4 %) | 42  (2.0 %) | 32  (1.5 %) |
| **Bulimia nervosa**  **N = 509** | 395  (77.6 %) | 67  (13.2 %) | 23  (4.5 %) | 18  (3.5 %) | 6  (1.2 %) |
| **Other / unspecified ED**  **N = 1613** | 1253  (77.7 %) | 193  (12.0 %) | 86  (5.3 %) | 42  (2.6 %) | 39  (2.4 %) |
| **Number of full siblings in control participants**  **(pooled according to the matched eating disorder group)** | | | | | |
|  | **0**  **N (%)** | **1**  **N (%)** | **2**  **N (%)** | **3**  **N (%)** | **4 or more**  **N (%)** |
| **Any ED**  **N = 16494** | 3603  (21.8 %) | 7226  (43.8 %) | 3873  (23.5 %) | 1037  (6.3 %) | 755  (4.6 %) |
| **Anorexia nervosa**  **N = 8233** | 1769  (21.5 %) | 3572  (43.4 %) | 2000  (24.3 %) | 521  (6.3 %) | 371  (4.5 %) |
| **Bulimia nervosa**  **N = 1990** | 465  (23.4 %) | 891  (44.8 %) | 438  (22.0 %) | 118  (6.0 %) | 78  (3.9 %) |
| **Other / unspecified ED**  **N = 6271** | 1369  (21.8 %) | 2763  (44.1 %) | 1435  (22.9 %) | 398  (6.4 %) | 306  (4.9 %) |
| **Number of half siblings in control participants**  **(pooled according to the matched eating disorder group)** | | | | | |
|  | **0**  **N (%)** | **1**  **N (%)** | **2**  **N (%)** | **3**  **N (%)** | **4 or more**  **N (%)** |
| **Any ED**  **N = 16494** | 13533  (82.1 %) | 1508  (9.14 %) | 796  (4.8 %) | 365  (2.2 %) | 292  (1.8 %) |
| **Anorexia nervosa**  **N = 8233** | 6763  (82.15 %) | 727  (8.8 %) | 406  (4.9 %) | 191  (2.3 %) | 146  (1.8 %) |
| **Bulimia nervosa**  **N = 1990** | 1623  (81.6 %) | 186  (9.4 %) | 94  (4.7 %) | 46  (2.3 %) | 41  (2.1 %) |
| **Other / unspecified ED**  **N = 6271** | 5147  (82.1 %) | 595  (9.5 %) | 296  (4.7 %) | 128  (2.0 %) | 105  (1.7 %) |

# **Supplementary Table 2.** Concordant neurodevelopmental diagnoses between sibling pairs.

Abbreviations: eating disorder: ED; attention-deficit/hyperactivity disorder: ADHD; autism spectrum disorder: ASD; tic disorder: TD; obsessive-compulsive disorder: OCD

|  |  | **Participants with ED** | **Control participants** | **Full siblings of ED participants** | **Full siblings of control participants** | **Half siblings of ED participants** | **Half siblings of control participants** |
| --- | --- | --- | --- | --- | --- | --- | --- |
| Total N |  | 4240 | 16494 | 5214 | 23641 | 1555 | 5891 |
| Neurodevelopmental diagnoses | ASD N (%) | 109  (2.6 %) | 58  (0.4 %) | 75  (1.5 %) | 164  (0.7 %) | 11  (0.7 %) | 35  (0.6 %) |
|  | Concordant ASD in sibling pair N (%) | <15  (< 13.8 %) | 7  (12.0 %) | 10  (13.3 %) | 7  (4.3 %) | <5  (< 45.5 %) | 0  (0.0 %) |
|  | ADHD N (%) | 117  (2.8 %) | 161  (1.0 %) | 103  (2.0 %) | 305  (1.3 %) | 34  (2.2 %) | 121  (2.1 %) |
|  | Concordant ADHD in sibling pair  N (%) | <18  (15.4 %) | 20  (12.4 %) | 13  (12.6 %) | 14  (4.6 %) | < 5  (14.7 %) | 7  (5.8 %) |
|  | TD N (%) | 22  (0.5 %) | 35  (0.2 %) | 26  (0.5 %) | 76  (0.3 %) | 5  (0.3 %) | 18  (0.3 %) |
|  | Concordant TD in sibling pair  N (%) | 0  (0.0 %) | < 5  (< 14.3 %) | 0  (0.0 %) | < 5  (< 6.6 %) | 0  (0.0 %) | 0  (0.0 %) |
|  | OCD N (%) | 157  (3.7 %) | 76  (0.5 %) | 30  (0.6 %) | 48  (0.2 %) | < 5  (< 0.3 %) | 11  (0.2 %) |
|  | Concordant OCD in sibling pair N (%) | < 5  (< 3 %) | < 5  (< 6.6 %) | < 5  (< 16.7 %) | < 5  (< 10.4 %) | 0  (0.0 %) | 0  (0.0 %) |

# **Supplementary Table 3.** Numbers of autism spectrum disorder (ASD) diagnoses in individuals with an eating disorder (ED) and in their full siblings and half siblings and in population controls and their siblings. If there are more than 0 but fewer than 5 individuals in specific category, this is marked as <5 to protect privacy.

| **All participants** | | | | | | | |
| --- | --- | --- | --- | --- | --- | --- | --- |
|  |  | **Cases** | | **Full siblings** | | **Half siblings** | |
| **ED diagnoses / pooled ED category** |  | **Total N** | **ASD N (%)** | **Total N** | **ASD N (%)** | **Total N** | **ASD N (%)** |
| **Anorexia nervosa**  **(F50.0, F50.1)** | Index individuals and their siblings | 2118 | 51 (2.4 %) | 2553 | 36 (1.4 %) | 643 | 7 (1.1 %) |
|  | Control individuals and their siblings | 8233 | 30 (0.4 %) | 11319 | 79 (0.7 %) | 2864 | 18 (0.6 %) |
| **Bulimia nervosa (F50.2, F50.3)** | Index individuals and their siblings | 509 | < 5 (< 1.0 %) | 570 | < 5 (< 0.9 %) | 195 | < 5 (< 2.6 %) |
|  | Control individuals and their siblings | 1990 | < 5 (< 0.3 %) | 2631 | 21 (0.8 %) | 709 | < 5 (< 0.7 %) |
| **Other /unspecified EDs**  **(F50.8, F50.9)** | Index individuals and their siblings | 1613 | 54 (3.4 %) | 2091 | 37 (1.8 %) | 717 | < 5 (< 0.7 %) |
|  | Control individuals and their siblings | 6271 | 24 (0.4 %) | 9691 | 64 (0.7 %) | 2318 | 14 (0.6 %) |
| **Females** | | | | | | | |
|  |  | **Cases** | | **Full siblings** | | **Half siblings** | |
| **ED diagnoses / pooled ED category** |  | **Total N** | **ASD N (%)** | **Total N** | **ASD N (%)** | **Total N** | **ASD N (%)** |
| **Anorexia nervosa**  **(F50.0, F50.1)** | Index individuals and their siblings | 1939 | 34 (1.8 %) | 1258 | 12 (1.0 %) | 312 | 0 (0.0 %) |
|  | Control individuals and their siblings | 7537 | 26 (0.3 %) | 5518 | 14 (0.3 %) | 1393 | < 5 (< 0.4 %) |
| **Bulimia nervosa (F50.2, F50.3)** | Index individuals and their siblings | 488 | < 5 (< 1.0 %) | 271 | 0 (0.0 %) | 96 | 0 (0.0 %) |
|  | Control individuals and their siblings | 1907 | < 5 (< 0.3 %) | 1280 | 6 (0.5 %) | 357 | < 5 (< 1.4 %) |
| **Other /unspecified EDs**  **(F50.8, F50.9)** | Index individuals and their siblings | 1387 | 29 (2.1 %) | 1070 | 6 (0.6 %) | 366 | < 5 (< 1.4 %) |
|  | Control individuals and their siblings | 5384 | 21 (0.4 %) | 4689 | 13 (0.2 %) | 1143 | < 5 (< 0.4 %) |
| **Males** | | | | | | | |
|  |  | **Cases** | | **Full siblings** | | **Half siblings** | |
| **ED diagnoses / pooled ED category** |  | **Total N** | **ASD N (%)** | **Total N** | **ASD N (%)** | **Total N** | **ASD N (%)** |
| **Anorexia nervosa**  **(F50.0, F50.1)** | Index individuals and their siblings | 179 | 17 (9.5 %) | 1295 | 24 (1.9 %) | 331 | 7 (2.1 %) |
|  | Control individuals and their siblings | 696 | < 5 (< 0.7 %) | 5801 | 65 (1.1 %) | 1471 | 16 (1.1 %) |
| **Bulimia nervosa (F50.2, F50.3)** | Index individuals and their siblings | 21 | < 5 (< 23.8 %) | 299 | < 5 (< 1.6 %) | 99 | < 5 (< 5.0 %) |
|  | Control individuals and their siblings | 83 | 0 (0.0 %) | 1351 | 15 (1.1 %) | 352 | < 5 (< 1.4 %) |
| **Other /unspecified EDs**  **(F50.8, F50.9)** | Index individuals and their siblings | 226 | 25 (11.1 %) | 1021 | 31 (3.0 %) | 351 | < 5 (< 1.4 %) |
|  | Control individuals and their siblings | 887 | < 5 (< 0.6 %) | 5002 | 51 (1.0 %) | 1175 | 12 (1.0 %) |

# **Supplementary Table 4.** Numbers of attention-deficit/hyperactivity disorder (ADHD) diagnoses in individuals with an eating disorder (ED) and in their full siblings and half siblings and in population controls and their siblings. If there are more than 0 but fewer than 5 individuals in specific category, this is marked as <5 to protect privacy.

| **All participants** | | | | | | | |
| --- | --- | --- | --- | --- | --- | --- | --- |
|  |  | **Cases** | | **Full siblings** | | **Half siblings** | |
| **ED diagnoses / pooled ED category** |  | **Total N** | **ADHD N (%)** | **Total N** | **ADHD N (%)** | **Total N** | **ADHD N (%)** |
| **Anorexia nervosa**  **(F50.0, F50.1)** | Index individuals and their siblings | 2118 | 59 (2.8 %) | 2553 | 31 (1.2 %) | 643 | 16 (2.5 %) |
|  | Control individuals and their siblings | 8233 | 71 (0.9 %) | 11319 | 135 (1.2 %) | 2864 | 57 (2.0 %) |
| **Bulimia nervosa (F50.2, F50.3)** | Index individuals and their siblings | 509 | 9 (1.8 %) | 570 | 14 (2.5 %) | 195 | 5 (2.6 %) |
|  | Control individuals and their siblings | 1990 | 19 (1.0 %) | 2631 | 41 (1.6 %) | 709 | 14 (2.0 %) |
| **Other /unspecified EDs**  **(F50.8, F50.9)** | Index individuals and their siblings | 1613 | 49 (3.0 %) | 2091 | 58 (2.8 %) | 717 | 13 (1.8 %) |
|  | Control individuals and their siblings | 6271 | 71 (1.1 %) | 9691 | 129 (1.3 %) | 2318 | 50 (2.2 %) |
| **Females** | | | | | | | |
|  |  | **Cases** | | **Full siblings** | | **Half siblings** | |
| **ED diagnoses / pooled ED category** |  | **Total N** | **ADHD N (%)** | **Total N** | **ADHD N (%)** | **Total N** | **ADHD N (%)** |
| **Anorexia nervosa**  **(F50.0, F50.1)** | Index individuals and their siblings | 1939 | 44 (2.3 %) | 1258 | 15 (1.2 %) | 312 | 5 (1.6 %) |
|  | Control individuals and their siblings | 7537 | 51 (0.7 %) | 5518 | 14 (0.3 %) | 1393 | 10 (0.7 %) |
| **Bulimia nervosa (F50.2, F50.3)** | Index individuals and their siblings | 488 | 9 (1.8 %) | 271 | < 5 (< 1.8 %) | 96 | < 5 (< 5.2 %) |
|  | Control individuals and their siblings | 1907 | 19 (1.0 %) | 1280 | 8 (0.6 %) | 357 | < 5 (< 1.4 %) |
| **Other /unspecified EDs**  **(F50.8, F50.9)** | Index individuals and their siblings | 1387 | 29 (2.1 %) | 1070 | 17 (1.6 %) | 366 | < 5 (< 1.4 %) |
|  | Control individuals and their siblings | 5384 | 46 (0.9 %) | 4689 | 20 (0.4 %) | 1143 | 9 (0.8 %) |
| **Males** | | | | | | | |
|  |  | **Cases** | | **Full siblings** | | **Half siblings** | |
| **ED diagnoses / pooled ED category** |  | **Total N** | **ADHD N (%)** | **Total N** | **ADHD N (%)** | **Total N** | **ADHD N (%)** |
| **Anorexia nervosa**  **(F50.0, F50.1)** | Index individuals and their siblings | 176 | 15 (8.4 %) | 1295 | 16 (1.2 %) | 331 | 11 (3.3 %) |
|  | Control individuals and their siblings | 696 | 20 (2.9 %) | 5801 | 121 (2.1 %) | 1471 | 47 (3.2 %) |
| **Bulimia nervosa (F50.2, F50.3)** | Index individuals and their siblings | 21 | 0 (0.0 %) | 299 | 13 (4.4 %) | 99 | < 5 (< 5.0 %) |
|  | Control individuals and their siblings | 83 | 0 (0.0 %) | 1351 | 33 (2.4 %) | 352 | 12 (3.4 %) |
| **Other /unspecified EDs**  **(F50.8, F50.9)** | Index individuals and their siblings | 226 | 20 (8.9 %) | 1021 | 41 (4.0 %) | 351 | 10 (2.9 %) |
|  | Control individuals and their siblings | 887 | 25 (2.8 %) | 5002 | 109 (2.2 %) | 1175 | 41 (3.5 %) |

# **Supplementary Table 5.** Numbers of tic disorder (TD) diagnoses in individuals with an eating disorder (ED) and in their full siblings and half siblings and in population controls and their siblings. If there are more than 0 but fewer than 5 individuals in specific category, this is marked as <5 to protect privacy.

| **All participants** | | | | | | | |
| --- | --- | --- | --- | --- | --- | --- | --- |
|  |  | **Cases** | | **Full siblings** | | **Half siblings** | |
| **ED diagnoses / pooled ED category** |  | **Total N** | **TD N (%)** | **Total N** | **TD N (%)** | **Total N** | **TD N (%)** |
| **Anorexia nervosa**  **(F50.0, F50.1)** | Index individuals and their siblings | 2118 | 10 (0.5 %) | 2553 | 10 (0.4 %) | 643 | < 5 (< 0.8 %) |
|  | Control individuals and their siblings | 8233 | 21 (0.3 %) | 11319 | 41 (0.4 %) | 2864 | 7 (0.2 %) |
| **Bulimia nervosa (F50.2, F50.3)** | Index individuals and their siblings | 509 | < 5 (< 1.0 %) | 570 | < 5 (< 0.9 %) | 195 | 0 (0.0 %) |
|  | Control individuals and their siblings | 1990 | 5 (0.1 %) | 2631 | 10 (0.4 %) | 709 | < 5 < (0.7 %) |
| **Other /unspecified EDs**  **(F50.8, F50.9)** | Index individuals and their siblings | 1613 | 11 (0.7%) | 2091 | 15 (0.7 %) | 717 | < 5 (< 0.7 %) |
|  | Control individuals and their siblings | 6271 | 9 (0.1 %) | 9691 | 25 (0.3 %) | 2318 | 9 (0.4 %) |
| **Females** | | | | | | | |
|  |  | **Cases** | | **Full siblings** | | **Half siblings** | |
| **ED diagnoses / pooled ED category** |  | **Total N** | **TD N (%)** | **Total N** | **TD N (%)** | **Total N** | **TD N (%)** |
| **Anorexia nervosa**  **(F50.0, F50.1)** | Index individuals and their siblings | 1939 | 8 (0.4 %) | 1258 | < 5 (< 0.4 %) | 312 | 0 (0.0 %) |
|  | Control individuals and their siblings | 7537 | 20 (0.3 %) | 5518 | 9 (0.2 %) | 1393 | < 5 (< 0.4 %) |
| **Bulimia nervosa (F50.2, F50.3)** | Index individuals and their siblings | 488 | < 5 (< 1.0 %) | 271 | 0 (0.0 %) | 96 | 0 (0.0 %) |
|  | Control individuals and their siblings | 1907 | < 5 (< 0.3 %) | 1280 | < 5 (< 0.4 %) | 357 | 0 (0.0 %) |
| **Other /unspecified EDs**  **(F50.8, F50.9)** | Index individuals and their siblings | 1387 | < 5 (< 0.4 %) | 1070 | < 5 (< 0.5 %) | 366 | < 5 (< 1.4 %) |
|  | Control individuals and their siblings | 5384 | 5 (0.1 %) | 4689 | 8 (0.2 %) | 1143 | < 5 (< 0.4 %) |
| **Males** | | | | | | | |
|  |  | **Cases** | | **Full siblings** | | **Half siblings** | |
| **ED diagnoses / pooled ED category** |  | **Total N** | **TD N (%)** | **Total N** | **TD N (%)** | **Total N** | **TD N (%)** |
| **Anorexia nervosa**  **(F50.0, F50.1)** | Index individuals and their siblings | 176 | < 5 (< 0.3 %) | 1295 | 9 (0.7 %) | 331 | < 5 (< 1.5 %) |
|  | Control individuals and their siblings | 696 | < 5 (< 0.7 %) | 5801 | 32 (0.6 %) | 1471 | < 5 (< 0.3 %) |
| **Bulimia nervosa (F50.2, F50.3)** | Index individuals and their siblings | 21 | 0 (0.0 %) | 299 | < 5 (< 1.7 %) | 99 | 0 (0.0 %) |
|  | Control individuals and their siblings | 83 | < 5 (< 6.0 %) | 1351 | 7 (0.5 %) | 352 | < 5 (< 1.4 %) |
| **Other /unspecified EDs**  **(F50.8, F50.9)** | Index individuals and their siblings | 226 | 7 (3.1 %) | 1021 | 12 (1.2 %) | 351 | < 5 (< 1.4 %) |
|  | Control individuals and their siblings | 887 | < 5 (< 0.6 %) | 5002 | 17 (0.3 %) | 1175 | 7 (0.6 %) |

# **Supplementary Table 6.** Numbers of obsessive-compulsive disorder (OCD) diagnoses in individuals with an eating disorder (ED) and in their full siblings and half siblings and in population controls and their siblings. If there are more than 0 but fewer than 5 individuals in specific category, this is marked as <5 to protect privacy.

| **All participants** | | | | | | | |
| --- | --- | --- | --- | --- | --- | --- | --- |
|  |  | **Cases** | | **Full siblings** | | **Half siblings** | |
| **ED diagnoses / pooled ED category** |  | **Total N** | **OCD N (%)** | **Total N** | **OCD N (%)** | **Total N** | **OCD N (%)** |
| **Anorexia nervosa**  **(F50.0, F50.1)** | Index individuals and their siblings | 2118 | 86 (4.1 %) | 2553 | 18 (0.7 %) | 643 | < 5 (< 0.8 %) |
|  | Control individuals and their siblings | 8233 | 32 (0.4 %) | 11319 | 24 (0.2 %) | 2864 | < 5 (< 0.2 %) |
| **Bulimia nervosa (F50.2, F50.3)** | Index individuals and their siblings | 509 | 12 (2.4 %) | 570 | < 5 (< 0.9 %) | 195 | 0 (0.0 %) |
|  | Control individuals and their siblings | 1990 | 14 (0.7 %) | 2631 | < 5 (< 0.2 %) | 709 | 0 (0.0 %) |
| **Other /unspecified EDs**  **(F50.8, F50.9)** | Index individuals and their siblings | 1613 | 59 (3.7 %) | 2091 | 10 (0.5 %) | 717 | < 5 (< 0.7 %) |
|  | Control individuals and their siblings | 6271 | 30 (0.5 %) | 9691 | 20 (0.2 %) | 2318 | 9 (0.4 %) |
| **Females** | | | | | | | |
|  |  | **Cases** | | **Full siblings** | | **Half siblings** | |
| **ED diagnoses / pooled ED category** |  | **Total N** | **OCD N (%)** | **Total N** | **OCD N (%)** | **Total N** | **OCD N (%)** |
| **Anorexia nervosa**  **(F50.0, F50.1)** | Index individuals and their siblings | 1939 | 76 (3.9 %) | 1258 | 8 (0.6 %) | 312 | < 5 (< 1.6 %) |
|  | Control individuals and their siblings | 7537 | 32 (0.4 %) | 5518 | 10 (0.2 %) | 1393 | < 5 (< 0.4 %) |
| **Bulimia nervosa (F50.2, F50.3)** | Index individuals and their siblings | 488 | 12 (2.5 %) | 271 | < 5 (< 1.8 %) | 96 | 0 (0.0 %) |
|  | Control individuals and their siblings | 1907 | 13 (0.7 %) | 1280 | < 5 (< 0.4 %) | 357 | 0 (0.0 %) |
| **Other /unspecified EDs**  **(F50.8, F50.9)** | Index individuals and their siblings | 1387 | 49 (3.5 %) | 1070 | 7 (0.7 %) | 366 | 0 (0.0 %) |
|  | Control individuals and their siblings | 5384 | 25 (0.5 %) | 4689 | 12 (0.3 %) | 1143 | 7 (0.2 %) |
| **Males** | | | | | | | |
|  |  | **Cases** | | **Full siblings** | | **Half siblings** | |
| **ED diagnoses / pooled ED category** |  | **Total N** | **OCD N (%)** | **Total N** | **OCD N (%)** | **Total N** | **OCD N (%)** |
| **Anorexia nervosa**  **(F50.0, F50.1)** | Index individuals and their siblings | 176 | 10 (5.6 %) | 1295 | 10 (0.8 %) | 331 | < 5 (< 1.5 %) |
|  | Control individuals and their siblings | 696 | 0 (0.0 %) | 5801 | 14 (0.2 %) | 1471 | < 5 (< 0.3 %) |
| **Bulimia nervosa (F50.2, F50.3)** | Index individuals and their siblings | 21 | 0 (0.0 %) | 299 | 0 (0.0 %) | 99 | 0 (0.0 %) |
|  | Control individuals and their siblings | 83 | < 5 (< 0.6 %) | 1351 | < 5 (< 0.3 %) | 352 | 0 (0.0 %) |
| **Other /unspecified EDs**  **(F50.8, F50.9)** | Index individuals and their siblings | 226 | 10 (4.4 %) | 1021 | < 5 (< 0.5 %) | 351 | < 5 (< 1.4 %) |
|  | Control individuals and their siblings | 887 | 5 (0.6 %) | 5002 | 8 (0.2 %) | 1175 | < 5 (< 0.4 %) |
